# Phytochemical and Safety Evaluations of Finger Lime, Mountain Pepper, and Tamarind in Zebrafish Embryos

**DOI:** 10.3390/antiox11071280

**Published:** 2022-06-28

**Authors:** Paolin Rocio Cáceres-Vélez, Akhtar Ali, Alexandre Fournier-Level, Frank R. Dunshea, Patricia Regina Jusuf

**Affiliations:** 1 School of Biosciences, The University of Melbourne, Melbourne, VIC 3010, Australia; alexandre.fournier@unimelb.edu.au; 2 School of Agriculture and Food, The University of Melbourne, Melbourne, VIC 3010, Australia; akali@student.unimelb.edu.au (A.A.); fdunshea@unimelb.edu.au (F.R.D.); 3 Faculty of Biological Sciences, The University of Leeds, Leeds LS2 9JT, UK

**Keywords:** zebrafish, plant toxicity screening, phytochemical characterization, antioxidant capacity, native Australian plants, finger lime, mountain pepper, tamarind

## Abstract

Plants play a pivotal role in drug discovery, constituting 50% of modern pharmacopeia. Many human diseases, including age-related degenerative diseases, converge onto common cellular oxidative stress pathways. This provides an opportunity to develop broad treatments to treat a wide range of diseases in the ageing population. Here, we characterize and assess the toxicological effects of finger lime (*Citrus australasica*), mountain pepper (*Tasmannia lanceolata*), and small-leaved tamarind (*Diploglottis australis*) extracts. The characterization demonstrates that these Australian native plants have antioxidant potential and, importantly, they have high concentrations of distinct combinations of different antioxidant classes. Using zebrafish larvae as a high-throughput pre-clinical in vivo toxicology screening model, our experiment effectively discriminates which of these extracts (and at what exposure levels) are suitable for development towards future therapies. The LC50-96h for finger lime and tamarind were >480 mg/L, and 1.70 mg/L for mountain pepper. Critically, this work shows that adverse effects are not correlated to the properties of these antioxidants, thus highlighting the need for combining characterization and in vivo screening to identify the most promising plant extracts for further development. Thus, we present a high-throughput pre-clinical screening that robustly tests natural plant products to utilize the diversity of antioxidant compounds for drug development.

## 1. Introduction

Novel drugs sourced from natural flora are essential for developing new therapeutic approaches. The chemical compounds present in plants can be classified into primary or secondary metabolites based on their physiological role, chemical structure and biosynthetic derivation [1,2]. Primary metabolites support core cellular functions and include small molecules such as sugars, amino acids, and polysaccharides [2]. Secondary metabolites support peripheral functions in plant cells and are derived from primary metabolic pathways [2]. Secondary metabolites include terpenoids, phenolics, flavonoids, alkaloids, and glycosides. These secondary metabolites have formed the basis for treating many diseases, and act as an valuable source for bioactive ingredients in nutraceuticals and modern medicines [1,2,3]. Due to their therapeutic potential against a wide range of diseases, natural plant product drug discovery is an important step before envisioning translational development. As a megadiverse country, Australia has a unique flora setting with more than 25,000 endemic plant species [4]. Many of these contain unique combinations of metabolites that might act synergistically toward supporting cellular and human health. Three Australian plants were selected in this study, finger lime (*Citrus australasica*), mountain pepper (*Tasmannia lanceolata*), and tamarind (*Diploglottis australis*) (courtesy of Julie Weatherhead and Anthony Hooper, Peppermint Ridge Farm, Tynong North VIC 3813, Australia). Finger lime is a small tree found in the southern and northern regions of New South Wales, Australia [5,6]. The fruit is cylindrical, with colour ranging from green to pink. It is used for cooking and different preparations. Mountain pepper is a medium to large shrub, endemic to the rainforests of Tasmania and the south-eastern region of the Australian mainland [7]. The berries, leaves and bark of this species have historical uses as food and medicine [6,8]. Tamarind fruit has a high antioxidant capacity and has mainly been used as a nutritious food. The fruit has reported antibacterial and anticancer properties [9,10]. Despite its ethnobotanical usage and antioxidant properties [11], limited studies have assessed its therapeutic properties. Importantly, toxicological studies on these plants are scarce.

Many studies have focused on the antioxidant potential of natural compounds [12,13,14,15]. This is particularly relevant to the aging process, which is driven by a series of interrelated mechanisms, among which oxidative stress, inflammation status, and autophagy function through diverse signalling pathways. Among these, the potential anti-aging benefits of polyphenol antioxidants have gained increasing scientific interest due to their capacity to modulate oxidative damage and inflammation [12,13,16,17]. Although reactive oxygen species (ROS) production in cells is involved with normal function in cell signalling, excessive ROS build-up results in oxidative stress, leading to cellular damage [17,18]. Most ROS result from mitochondrial metabolism [19], and an imbalance between ROS generation and endogenous antioxidants can induce mitochondrial dysfunction and, consequently, cell death [19,20]. Thus, growing evidence suggests a crucial connection between ROS formation and age-related disorders, including cardiovascular, neurodegenerative diseases, and cancer [20,21]. In unique combinations, Australian plants produce natural antioxidant compounds [7,11,22] in both hydrophilic and lipophilic fractions [22]. These could be used to treat oxidative stress-related diseases in which mitochondrial metabolism imbalance plays a central role by stimulating pro-regenerative/survival factors. Even though interest in antioxidant compounds from native Australian plants has increased in the last decade [7,11,23], the benefits as well as the adverse effects of such compounds are yet to be evaluated systematically.

For drug discovery, the toxicology screening of plant extracts must first be completed to identify safe concentrations and minimize potential adverse effects for subsequent efficacy testing [24]. Here, we present a non-invasive, rapid high-throughput in vivo screening system to aid in such systematic evaluation. Zebrafish are an established vertebrate model suitable for chemical toxicological screening [25,26] and represent an important pre-clinical in vivo bridge between in vitro assays and mammalian in vivo studies [27,28]. Here, we use zebrafish to assess the safe concentrations and acute toxicity of finger lime, mountain pepper, and tamarind extracts as potential candidates for future antioxidant treatments. By systematically accumulating data on different native plants, we will be able to use this screening platform and subsequent antioxidant efficacy testing in the same model to start identifying useful synergistic combinations of compounds and dive further into the cellular pathways that drive the most beneficial cellular processes.

## 2. Materials and Methods

### 2.1. Materials

Extraction was performed using analytical-grade ethanol (Thermo Fisher Scientific Inc., Scoresby, VIC, Australia). For the quantification of polyphenols and antioxidant potential, Folin-Ciocalteu’s phenol reagent, gallic acid, L-ascorbic acid, vanillin, hexahydrate aluminium chloride, sodium phosphate, iron(III) chloride hexahydrate (Fe[III]Cl3·6H2O), sodium phosphate dibasic hepta-hydrate, sodium phosphate monobasic monohydrate, trichloroacetic acid, hydrated sodium acetate, hydrochloric acid, ethylenediaminetetraacetic acid (EDTA), ferrozine, iron (II) chloride, iron (III) chloride, 3-hydrobenzoic acid, ammonium molybdate, quercetin, catechin, iron (II) sulphate heptahydrate, DPPH, 2,4,6tripyridyl-s-triazine (TPTZ), potassium ferrocyanide (III), and ABTS were purchased from Sigma-Aldrich (Castle Hill, NSW, Australia). Sodium carbonate anhydrous and hydrogen peroxide (30%) were purchased from Chem-Supply Pty Ltd. (Adelaide, SA, Australia) and 98% sulfuric acid was purchased from RCI Labscan (Rongmuang, Thailand). For HPLC and LC-MS, analytical-grade reagents including methanol, ethanol, acetonitrile, formic acid, iron (III) chloride anhydrous and glacial acetic acid, as well as 96 well-plates, were purchased from Thermo Fisher Scientific Inc. (Scoresby, VIC, Australia). Standards for HPLC quantification were purchased from Sigma and HPLC vials (1 mL) were purchased from Agilent Technologies (Melbourne, VIC, Australia).

### 2.2. Preparation of Plant Extracts

Fresh finger lime fruits (*Citrus australasica*), mountain pepper leaf (*Tasmannia lanceolata*), and tamarind fruits (*Diploglottis australis*) were collected from Peppermint Ridge Farm, Melbourne, Australia. The plant material collected was stored at −80 °C, thawed prior to extraction, and blended to reduce the particle size and facilitate the extraction with the solvent. One gram of plant material was prepared by adding 10 mL of analytical-grade ethanol (70%). The bottles containing the samples were covered with aluminium foil and placed in a shaking incubator at 120 rpm at 4 °C for 24 h. The extracts were filtered using a paper filter to remove the particles in suspension. The solvent from the extracts was removed by lyophilization. The purified samples were stored at −80 °C and covered from light until use.

### 2.3. Determination of Total Phenolic Content (TPC) and Antioxidant Activity

Total phenolics and their antioxidant activities were estimated by following the methods of Ali et al. [29]. For TPC, a total of 50 μL of the extracted sample or standard and Folin-Ciocalteu (F-C) reagent (1:1 ratio) were mixed before 200 μL milli-Q water was added and the mixture was dark-incubated for 5 min. Then, 25 μL of 10% Na_2_CO_3_ was added and the absorbance at 765 nm was recorded after 1 h. Gallic acid (0–200 μg/mL) was used to generate the standard curve for the quantification of TPC. DPPH (modified from Chou et al. [30]) and ABTS (described by Bashmil et al. [31]) assays must be conducted in the dark (presence of red light). For DPPH, 275 μL (0.1 mM DPPH) and 25 μL sample or standard were mixed and incubated for 30 min in the dark before spectrophotometer reading at 517 nm. DPPH was measured by comparing the Trolox (0–100 μg/mL). For ABTS, after 16 h, absorbance was calibrated at 0.70 ± 0.02 and 10 μL sample or standard was mixed with 290 μL ABTS dye in the dark and incubated for 6 min at room temperature, and the calibration line was obtained by running Trolox (0–200 μg/mL) in methanol. For RPA, 50 μL solution of 0.2 M phosphate buffer and 1% potassium ferricyanide in the ratio of 1:1 (*v/v*) were mixed with 10 μL sample or standard and incubated for 20 min at room temperature, and then the reaction was stopped with 25 μL 10% TCA. Furthermore, 85 μL Milli Q water and 10 μL FeCl_3_ were added and incubated for 15 min at room temperature in the dark, and absorbance at 750 nm was recorded. RPA was measured by comparing the Trolox (0–600 μg/mL) as an external standard through the spectrophotometer. All experiments were performed in triplicate. 

### 2.4. Screening for Bioactive Compounds in Plant Extracts

Polyphenolic compounds were identified following Ali et al. [32] with modifications using the Agilent 6520 Accurate-Mass QTOF machine. Synergi Hydro-RP (4 μm particle size, 4.6 mm internal diameter, and 250 mm length with 80 Å pore size) was used for the separation of phenolic compounds and the flow rate was set at 600 μL/min. Ten microlitres of extract was injected while the gradient was set to 0–30 min (10–35% B), 30–35 min (35–40% B), 35–50 min (40–55% B), 50–60 min (55–75%), 60–70 min (75–90% B), 70–75 min (90–100% B), 75–77 min (100% B), 77–79 min (100–10% B), and 79–80 min (10% B) intervals. The mobile phase A was 0.1% formic acid in water and mobile phase B was 95% acetonitrile with 0.1% formic acid. A full scan mode was achieved in the range of 90–1300 amu with the following conditions: a capillary voltage (3.5 kV), nozzle voltage (500 V), and drying gas flow rate (9 L/min) at 325 °C; nebulization was set to 45 psi, and 10, 15 and 30 eV collision energies were used. Acquisition (4 spectra/second) was achieved in auto MS/MS positive and negative mode. MassHunter Workstation Software (version B.06.00, Santa Clara, CA, United States) was used for the extraction and identification of phenolic compounds. These experiments were completed in duplicate. 

### 2.5. Quantification of Individual Bioactive Antioxidant Compounds in Plant Extracts

The quantification of individual compounds was achieved by following the method of Sharifi-Rad et al. [33] with modifications. Agilent 1200 Series HPLC (Agilent, Santa Clara, CA, United States) equipped with a photodiode array (PDA) was used by following the same gradient, mobile phase and column as LCMS but injecting 20 μL of each extract. The detection wavelengths were set at 280 nm, 320 nm, and 370 nm by using the PDA detector with 1.25 scan/s (peak width = 2 min) spectra acquisition rate. Fifteen (15) compounds were quantified in triplicate on a dry weight basis (μg/g). 

### 2.6. In Vivo Acute Toxicity Test of Australian Plants

Zebrafish (*Danio rerio*) of the AB strain were raised and maintained at the Danio Rerio University of Melbourne facility (DrUM, Melbourne, Australia) in accordance with local animal guidelines and husbandry and breeding ethics. All in vivo experiments were conducted in compliance with the Australian code for the care and use of animals for scientific purposes and the regulation of the Animal welfare and Animal Ethics Committee of the University of Melbourne (Melbourne, Australia). Embryos were collected from breeding zebrafish adults into E3 medium (5 mM NaCl; 0.17 mM KCl; 0.33 mM CaCl_2_; 0.33 mM MgSO_4_). Fertilised eggs undergoing normal morphological development (i.e., “healthy”) within the first three hours post-fertilization were selected using a LEICA M80 stereomicroscope (Leica, Wetzlar, Germany).

For assessing the acute effects of the extracts, preliminary tests were carried out (data not shown) based on the Fish Embryo Acute Toxicity Test (OECD 236, 2013) [26]. For each plant extract, a stock solution was freshly prepared in autoclaved E3 medium and diluted to obtain increasing concentrations as follows: 15; 30; 60; 120; 240; and 480 mg/L for finger lime and tamarind; and 0.59; 0.84; 1.2; 1.72; 2.45; and 3.5 mg/L for mountain pepper; or E3 only for the controls. At 3 hpf, zebrafish embryos were exposed in a static system as described by Caceres-Velez et al. [34]. Briefly, healthy embryos were pre-exposed to their relevant concentration and then placed into 46-well plates (one embryo/well) containing 500 μL/well of test solution. Three independent replicates were tested for each treatment with a total of 60 embryos per concentration of each compound. For each fish, daily (24, 48, 72, and 96 h of exposure) phenotypic and behavioural endpoints were evaluated using a stereomicroscope (LEICA M80, Leica, Wetzlar, Germany). These included: (a) signs of mortality (coagulation, dead embryo/larvae) and morbidity (heart beating, no blood circulation in the tail, and balance disorder); (b) alterations (hatching, pigmentation and yolk sac absorption delays, cardiac and yolk sac edema); and (c) malformations (in the head, eyes, spine, tail, and somites). Balance disorder was considered as a behavioural trait. 

### 2.7. Statistical Analysis

The data were analysed by one-way analysis of variance (ANOVA) followed by Dunnett’s multiple comparisons test, and a Probit model was fitted for determining the LC50. GraphPad Prism 9.3.0 (GraphPad Software, San Diego, CA, USA) was used with the level of significance set at 5%.

## 3. Results and Discussion

### 3.1. Characterization of Australian Native Plant Extracts

#### 3.1.1. Total Phenolic Content and Antioxidant Activity

The chosen native Australian plants showed a distinct combination of antioxidants, as measured through the TPC, DPPH, ABTS and RPA assays (Table 1). Overall, higher antioxidant activity was found in mountain pepper and tamarind while the least antioxidant activity was observed in finger lime.

Mountain pepper showed the highest TPC (5.91 ± 0.32 mg GAE/g) in this study and finger lime, the lowest (0.71 ± 0.00 mg GAE/g). Finger lime total phenolics were previously measured by Konczak and colleagues (2010) [35] in the range of 6.8–9.2 μm/g (0.0068–0.0092 mg/g) FW and quantified by Sommano and colleagues (2013) [36] as 4.57 mg/g antioxidant. For tamarind, Shlini and Siddalinga (2011) [37] previously quantified 1.4 mg/g total phenolics. These values are very similar to those obtained in one of our other studies, where extraction with 80% methanol and 1% formic acid in finger lime and mountain pepperberry showed TPC 3.86 ± 0.51 mg GAE/g and 4.10 ± 0.34 mg GAE/g, respectively (data not published). These values are also comparable with related plants we analysed previously, where the quantification of total polyphenols in black pepper, black cardamom, black cumin, and green cardamom yielded 8.06 ± 0.08, 5.54 ± 0.19, 4.02 ± 0.23, and 3.30 ± 0.32 mg GAE/g, respectively [29]. Berries are generally recognized as being excellent sources of antioxidants, and mountain pepper and tamarind had 2 to 3 times higher TPC and antioxidant concentrations than Australian-grown blueberries, strawberries, blackberries, and raspberries [38].

DPPH and ABTS are commonly used assays for the measurement of the total antioxidant potential of plant extracts. The highest DPPH value was found in mountain pepper (4.48 ± 0.03 mg/g), while the lowest value was found in finger lime (0.44 ± 0.06 mg/g). The same trend was observed for RPA and ABTS. Sommano et al. (2013) [37] previously reported the 28.46 TE mg/100g Trolox equivalent antioxidant capacity (TEAC) of finger lime, which is in the same order of magnitude as our values.

Different in vitro assays can be applied to quantify the targeted antioxidant potential of these plants. These data highlight the difference between these values across studies, which could be due to extraction conditions (solvent type and ratio, solvent concentration, time and temperature), plant genotypes, and growth conditions (temperature, moisture, and edaphic factors), which may influence the phenolic content in the plants [39], as previously reported by Ali et al. [32]. This emphasizes the need to perform such characterization for every batch of plants used for further toxicology and efficacy testing. As an example, TPC and antioxidant concentrations vary widely across varieties, stage of ripening, and the part of the fruit (peel versus pulp) of bananas [31], and it is widely known that the method of extraction can markedly affect the estimation of antioxidant concentrations [40]. 

#### 3.1.2. Identification of Individual Bioactive Antioxidant Compounds in Plant Extracts

The untargeted characterization and identification of these targeted antioxidant bioactive metabolites of plants extracts was attained using HPLC-ESI-QTOF-MS/MS in positive and negative mode (Figures S1 and S3). Our analysis identified a total of 96 bioactive metabolites that were confirmed by fragment mass spectra using the personal compound database and library (PCDL) for metabolites, online libraries, and published literature (Table S1). These included 25 phenolic acids, 44 flavonoids, 19 other polyphenols, 3 stilbenes, 2 lignans, 1 sesquiterpenoids, and 2 other non-phenolic metabolites. The highest number of bioactive metabolites (62) were identified in mountain pepper, while 44 and 32 bioactive metabolites were identified in tamarind and finger lime, respectively. 

##### Phenolic Acids

Due to their antioxidant potential and associated health benefits, phenolic metabolites have gained much interest in recent decades in drug discovery [17]. Phenolic acids are a highly diversified group of aromatic, non-flavonoid secondary metabolites. They are present in all land plants in free, soluble, insoluble, conjugated, or bound forms. Phenolic acids with C1-C6 and C3-C6 backbones are classified into hydroxybenzoic and hydroxycinnamic acids. As they represent ubiquitous constituents/compounds of plants that have raised interest due to their potential health effects [41], we directly assessed their abundance in our samples. A total of 25 phenolic acids (a similar range is seen across botanicals) were identified based on their MS/MS spectra (see Table S1 for a comprehensive list and further detail, including mode of ionization, *m/z*, fragment, and sample detected in). Well-known phenolic acids encountered at relatively high abundance in our samples included chlorogenic acid, gallic acid, and 2-hydroxybenzoic acid, which were all identified and quantified in finger lime, mountain pepper, and tamarind. Caffeic acid, previously identified in tamarind seeds by El-Haddad et al. [42], was also identified in all three samples. Furthermore, cinnamic acid (previously identified in star anise [30]) was identified in mountain pepper and tamarind (Figure S2), and ferulic acid was detected in mountain pepper and finger lime (Figure S2). These phenolics have been specifically highlighted as they are recognized for their medicinal properties [43,44].

##### Flavonoids

Flavonoids are the largest group of phenolic compounds with antioxidant, anti-inflammatory, anti-cancer, anti-mutagenic, and anti-diabetic properties [19,29]. In this study, a total of 44 flavonoids were identified in the native Australian plants tested (see Table S1 for a comprehensive list and further detail, including mode of ionization, *m/z*, fragment, and sample detected in). The following flavonoids were common to all three of our samples: catechin, luteolin, procyanidin dimer B2, procyanidin trimer, and procyanidin tetramer. These were also detected by Sudjaroen et al. [45] in tamarind, as well as apigenin 6-8-di-C-glucoside (C_27_H_30_O_15_), and myricetin 3-*O*-rhamnoside (C_21_H_20_O_12_). Quercetin 3-*O*-arabinoside and quercetin 3′-*O*-glucuronide were only identified in mountain pepper and finger lime, and 3′-hydroxydaidzein was only identified in mountain pepper and tamarind. These flavonoids have been reported for their potent biological activities [46,47,48,49,50].

##### Other Biologically Relevant Compounds Identified

A number of other subclasses of compounds have been described as having particularly potent health effects, including as antioxidants. Of these, our samples contained a great variety of individual compounds, the common ones of which are described here. Anthocyanins are the main red-to-purple pigments in plants [51] and have been shown to have antioxidant, anti-cancer, neuro- and cardioprotective and anti-diabetic health potential [52]. Excitingly, a total of eight anthocyanins were identified in our samples, with one in Australian native finger lime, two in mountain pepper, and seven in tamarind (Table S1).

Lignans and stilbenes are also subclasses of phenolic compounds with substantial antioxidant, anti-carcinogenic, and anti-inflammatory activity [32]. In this study, through LC-MS/MS-QTOF (Table S1), a total of four lignans and stilbenes were tentatively identified in mountain pepper, whereas a fifth stilbene was encountered only in finger lime and tamarind. Of particular interest, mountain pepper contained 3,4,5,4′-Tetramethoxystilbene, a resveratrol analog [53] which has been reported as an anti-cancer bioactive compound, and dihydroresveratrol, both of which are likely to afford positive health effects. Curcumenol is a sesquiterpene tentatively identified in mountain pepper which has strong antioxidant, anti-inflammatory, anti-tumour, neuro-protective, and hepatoprotective activities [54]. Additionally, a total of 19 other polyphenols were identified in finger lime (4), mountain pepper (11) and tamarind (10) (Table S1). Esculin is a hydroxycoumarin identified in mountain pepper. Coumarin and umbelliferone were detected in mountain pepper and tamarind. Coumarins and derivatives are a widespread group in plants which have a number of health benefits [55,56].

Overall, our native plant extracts contained many bioactive compounds, including those that have previously been identified to carry a variety of potent health benefits, and also other related compounds belonging to the same subclasses. Thus, the LC-MS/MS-QTOF employed here remains a powerful analytical tool to identify unknown bioactive compounds and their relative abundance and unique combinations within different plant species for development in drug science. The in-depth characterization and identification of these compounds in complex biological samples will be critical to further establish scientific approaches for the separation and purification of these compounds to test their individual and synergistic biological potentials.

#### 3.1.3. Targeted Quantification of Bioactive Metabolites in Plant Extracts

The quantification of targeted bioactive metabolites was achieved by peak area computation and the results are reported in μg/g in Table S2. A total of 20 compounds were targeted, from which 15 compounds were quantified in finger lime, mountain pepper and tamarind. These included phenolic acids (nine), flavonoids (four), stilbenes (one), and other polyphenols (one). Phenolic acids, present in all samples, were the most abundant, and were also the class with the most described health benefits, suggesting that they might provide the most relevant antioxidant activity for therapeutic benefit.

By far the most abundant and present in all three samples were quinic acid derivatives (5-caffeoylquinic acid, 1,5-dicaffeoylquinic acid, and 3-feruloylquinic acid), suggesting that they might carry the greatest contribution to the total antioxidant activity quantified as shown above. 5-Caffeoylquinic acid (compound 3) was the most abundant phenolic acid in finger lime (132.91 ± 15.95 μg/g), mountain pepper (654.41 ± 43.44 μg/g) and tamarind (327.81 ± 54.81 μg/g). Previously, Sakulnarmrat and Konczak [57] also quantified 5-caffeoylquinic acid in a highly purified mountain pepper leaf (also called Tasmanian peppercorn). Gallic acid was quantified in mountain pepper (21.80 ± 2.24 μg/g) and tamarind (106.87 ± 14.93 μg/g), respectively. 1,5-Dicaffeoylquinic acid (134.73 ± 9.02 μg/g), 3-feruloylquinic acid (123.38 ± 5.63 μg/g), catechin 3-glucoside (15.88 ± 1.44 μg/g) and carvacrol (15.10 ± 0.05 μg/g) were only found in mountain pepper while pyrogallol (35.14 ± 8.73 μg/g) and PCB2 (41.02 + 5.93 μg/g) were only identified and quantified in tamarind. The highest concentration of catechin was quantified in tamarind (141.75 ± 9.29 μg/g), while catechin in the range of 18–22 μg/g was also found in mountain pepper and finger lime. Moreover, luteolin was quantified in mountain pepper (15.62 ± 1.34 μg/g) and tamarind (47.20 ± 8.69 μg/g). Catechin, procyanidin B2 and luteolin were also quantified by Sudjaroen et al. [45] in tamarind. Gallic acid, catechin, caffeic acid, coumaric acid, and cinnamic acid in tamarind were also quantified by El-Haddad et al. [42]. The described variation in values between our study and previous studies might be due to differences in genotypes, growth condition, or extraction method used, thus highlighting the importance of characterizing each sample to be correlated to any in vivo screening experiments.

**Figure 1:**
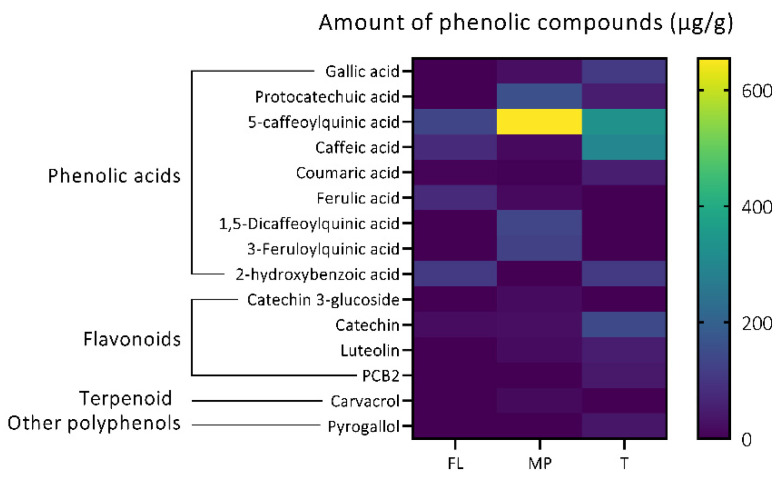
Heatmap of the concentration of bioactive compounds (μg/g) in finger lime (FL), mountain pepper (MP), and tamarind (T).

The profile and comparative abundance of phenolic compounds quantified in mountain pepper, finger lime, and tamarind are represented in Figure 1. Phenolic acids were the most abundant group quantified in this study. A total of nine phenolic acids were quantified, from which 5-caffeoylquinic acid (chlorogenic acid) was found with a higher concentration in mountain pepper (MP) compared to other metabolites. Caffeic acid was found in a higher concentration in tamarind. Furthermore, flavonoids (four), terpenoids (one) and other polyphenols (one) were also quantified in this study.

### 3.2. In Vivo Acute Toxicity of Australian Plants

**Figure 2:**
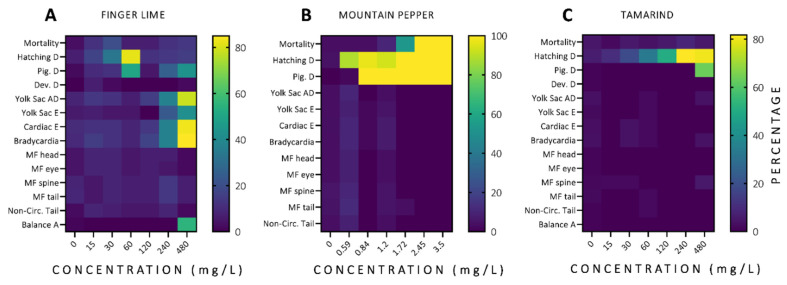
Heatmap of the alterations observed in zebrafish embryos exposed during 96 h to the plant extracts. The mean of three replicates (*n* = 20/replicate) is reported for finger lime (**A**), mountain pepper (**B**), and tamarind (**C**).

In order to estimate safe concentrations of finger lime, mountain pepper, and tamarind using an in vivo model, toxicological tests were performed during the embryo-larva development of zebrafish. Figure 2 shows the total adverse phenotypic and behavioural effects observed during 96 h of exposure for all plant extracts tested. The adverse effects observed with a frequency ≥80% of all tests conducted were hatching, yolk sac absorption delay, cardiac edema, and bradycardia for the zebrafish exposed to finger lime extract; mortality, hatching, and pigmentation delay for the mountain pepper group; and hatching delay for the tamarind group.

#### 3.2.1. Mortality and Morbidity

**Figure 3:**
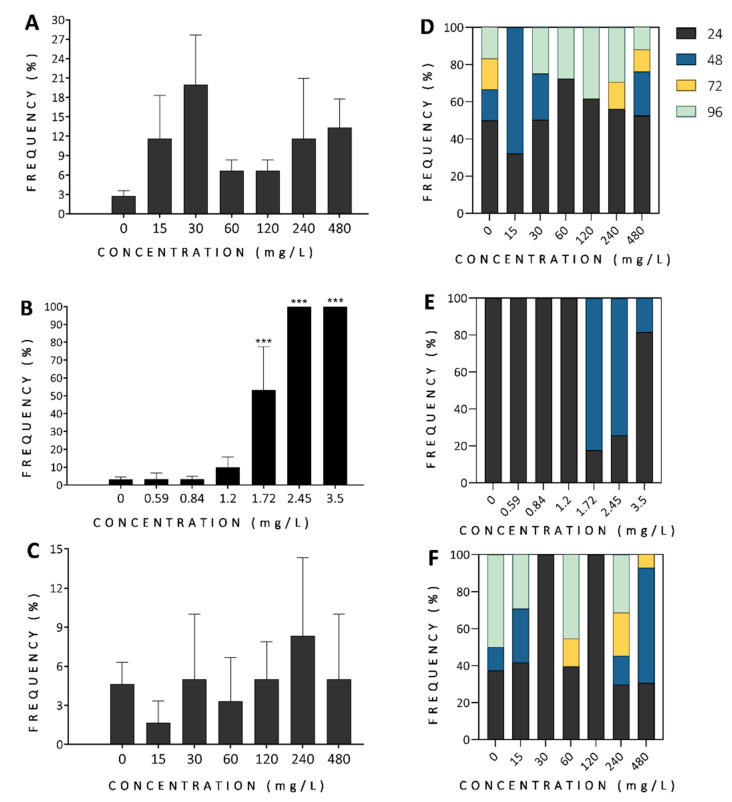
Phenotypic assessment of zebrafish development for the whole organism exposed 96 h post-fertilization to different concentrations of finger lime (**A**,**D**), mountain pepper (**B**,**E**), and tamarind (**C**,**F**). Cumulative mortality observed at 96 h of exposure are shown in panel (**A**) for finger lime, (**B**) for mountain pepper, and (**C**) for tamarind. The mortality frequency observed at different experimental times of exposure is shown for finger lime (**D**), mountain pepper (**E**), and tamarind (**F**). The colours in (**D**–**F**) represent the age at which mortality was observed (in hours post-fertilization). Data show the mean ± SD, *n* = 60. Asterisks indicate statistical significance when compared to control groups: *p* < 0.001 (***).

The control groups (0 mg/L) showed normal morphology and low mortality ≤5% over the complete experiment. The LC50-96h were calculated by scoring for egg coagulation (comprised from time (0–24 h) of exposure), dead embryos (before hatching, 24–48 h of exposure) and dead larvae (after hatching, 48–96 h of exposure), which were summarized as mortality (Figure 3). For finger lime and tamarind, the LC50-96h values were higher than the maximum concentration tested (480 mg/L) and for mountain pepper the LC50-96h was 1.70 mg/L. When mortality was analysed per day, the majority of embryos died during the first 24 h of exposure for finger lime for all the concentrations tested, except for 15 mg/L, where mortality peaked 48 h after exposure (Figure 3D). For mountain pepper, the mortality was concentration-dependent and lethality was primarily observed through egg coagulation (at 24 hpf) at concentrations of 0–1.2 mg/L and 3.5 mg/L (Figure 3E, *p* ≤ 0.001); and dead embryos (at 48 hpf) at 1.72 (*p* < 0.001) and 2.45 mg/L (*p* < 0.001). Although tamarind did not lead to more than 10% mortality for all concentrations tested, it was possible to identify, as shown in Figure 3C, that egg coagulation was predominant at 30, 120, and 240 mg/L; dead embryo was predominant at 480 mg/L; and dead larvae at 0 and 60 mg/L. Thus, mountain pepper extract showed the highest toxicity overall. Much of the observed mortality due to exposure to these plant extracts occurred mainly during the first 24 h of exposure, which encompasses the critical early developmental period in zebrafish embryo development. This was particularly true for mountain pepper and finger lime, although in the tamarind-exposed groups a second wave of mortality seemed to occur during 96 h of exposure, which could represent accumulated welfare issues.

**Figure 4:**
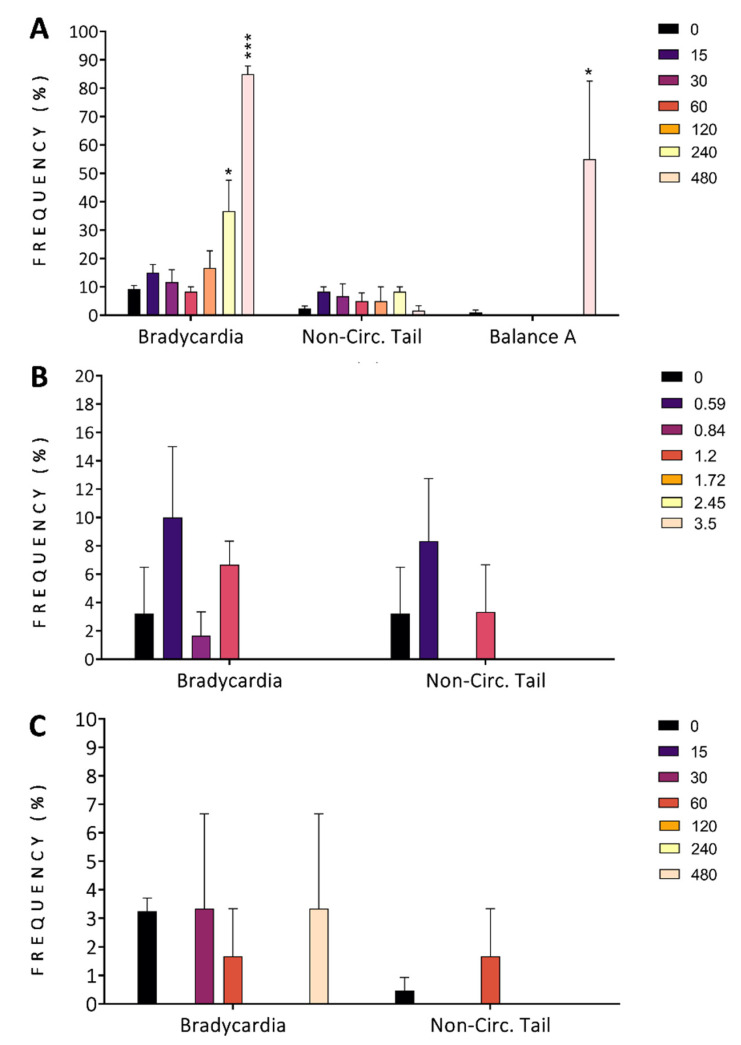
Morbidity assessments of zebrafish development for the whole organism exposed at 96 h post-fertilization to different concentrations of finger lime (**A**), mountain pepper (**B**), and tamarind (**C**). In (**C**), bradycardia is represented for groups exposed to tamarind. Non-Cir. Tail = non-circulation (of blood) in the tail; Balance A = balance alteration. Colours in (**A**,**B**) represent the concentrations tested (mg/L). Data represent the mean ± SD, *n* = 60. Asterisks indicate statistical significance when comparing the exposed groups with the control groups: *p* < 0.05 (*) and *p* < 0.001 (***).

Proxies for morbidity (bradycardia, non-circulation in the tail, and balance alteration) were observed at 96 h of exposure and are reported in Figure 4. Finger lime exhibited a concentration-dependent effect (CE) on bradycardia (240 and 480 mg/L, Figure 4A) and balance alteration (480 mg/L, Figure 4A), showing statistically significant differences (*p* < 0.05) across the concentrations tested. Non-circulation in the tail did not have a CE in the finger lime groups. When exposed to mountain pepper (Figure 4B) and tamarind (Figure 4C), no CE was observed for bradycardia and non-circulation in the tail.

#### 3.2.2. Developmental Alterations

**Figure 5:**
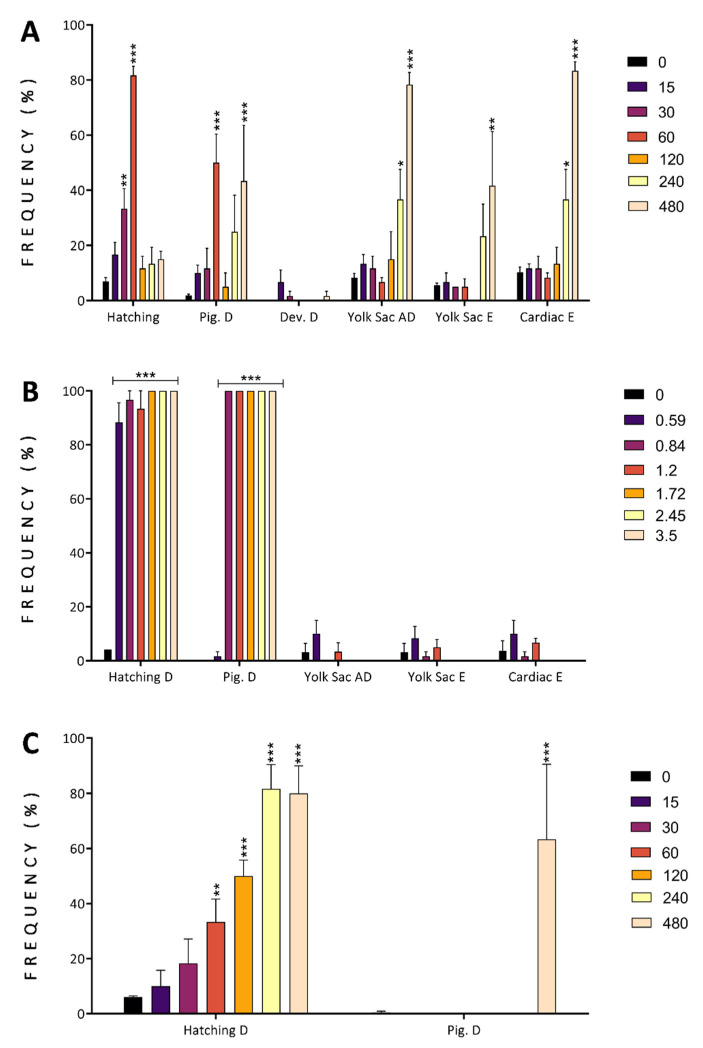
Developmental alterations observed in organisms exposed at 96 h post-fertilization to different concentrations of finger lime (**A**), mountain pepper (**B**), and tamarind (**C**) extracts. The alterations observed were hatching delay (Hatching D), pigmentation delay (Pig. D), developmental delay (Dev. D), yolk sac absorption delay (Yolk Sac AD), yolk sac edema (Yolk Sac E), and cardiac edema (Cardiac E). Bar colours represent the concentration tested in mg/L. Data represent the mean ± SD, *n* = 60. Asterisks indicate statistical significance when comparing the exposed groups with the control groups: *p* < 0.05 (*), *p* < 0.01(**) and *p* < 0.001 (***).

Zebrafish exposed to finger lime exhibited CE on yolk sac absorption delay, and yolk sac and cardiac edema (Figure 5A), while no CE was observed for balance, hatching, or pigmentation delays. The mountain pepper- and tamarind-exposed groups showed some similarities: there was a CE for hatching delay, but no CE for pigmentation delay (Figure 5B,C). However, the concentrations showing a significant effect were different for the two plants: hatching delay for mountain pepper was statistically significant (*p* < 0.001) from the lowest concentration tested (0.59 mg/L), while for tamarind, it was significant from 60 mg/L (*p* < 0.01). For pigmentation delay, the effect was similar (*p* < 0.001) from concentrations ≥ 0.84 for mountain pepper and 480 mg/L for tamarind. 

#### 3.2.3. Malformations

**Figure 6:**
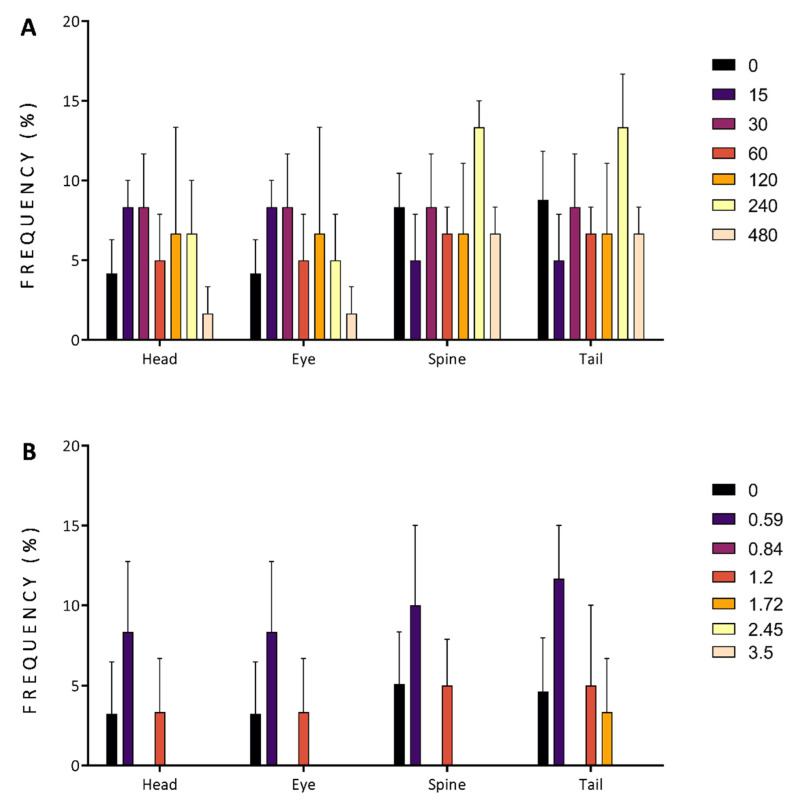
Malformations observed in zebrafish exposed at 96 h post-fertilization to different concentrations of finger lime and mountain pepper extracts. Each colour bar denotes the concentration tested in mg/L for finger lime (**A**) and mountain pepper (**B**). Malformations were mainly observed in the head, eyes, spine and tail. No statistically significant difference was observed when comparing the exposed and control groups for both finger lime and mountain pepper, and no malformations were observed for tamarind. Data represent the mean ± SD, *n* = 60.

Head, eye, spine, and tail malformations showed no statistical differences compared to the control groups for the finger lime (Figure 6A) and mountain pepper extracts (Figure 6B). For tamarind groups, these malformations were not observed through this acute toxicological assessment.

Despite the promising antioxidant capacity of the phytochemical profiles of finger lime, mountain pepper, and tamarind extracts [5,7,58,59], there is limited understanding of their potential toxicological effects in live vertebrate models. We comprehensively assessed the effects of plant extracts from these plants on zebrafish, a high-throughput in vivo model with ~75% genetic homology and high physiological and behavioural similarities to humans [60,61].

There are few toxicological in vitro studies published on finger lime [5] and mountain pepper [7,23,58,62], and no published in vitro and in vivo studies to date on tamarind. Shami et al., 2013, studied the antibacterial activity and antioxidant capacity of a mix of plants containing *Backhousia citriodora*, *Terminalia ferdinandiana*, *Citrus australasica* (finger lime) and *Lophopyrum ponticum,* commonly known as Australian wheatgrass sprouts [5]. In that study, they were able to describe that the ethanolic and peptidic plant extracts had high/efficient (not good) antibacterial activity against *S. aureus*, *E. coli,* and *B. cereus* [5]. However, the plant compounds were not tested individually, and further studies are needed to isolate and identify bioactive compounds in the mixture that could be used as sources to develop novel biopharmaceuticals against infectious diseases. 

Winnett et al. (2014) studied the potential of mountain pepper to block microbial and fungal food spoilage, indicating its therapeutic potential against infectious disease [7]. Rayan et al. (2017) described that methanolic, aqueous and ethyl acetate mountain pepper berry and leaf extracts displayed effective *G. duodenalis* growth inhibitory activity [62]. These authors found that methanolic extracts were the most potent growth inhibitors with IC50 values of approximately 180 µg/mL and 420 µg/mL for the berry and leaf methanolic extracts, respectively. Similarly, Wright et al. (2017) and Aldosary et al. (2019) have demonstrated the effectiveness of different mountain pepper extracts against food-poisoning pathogens, such as *C. perfringens* [58] and *Y. enterocolitica* [23]. The anti-proliferative activity against these microorganisms could be explained by the combination of phenolic acids and flavonoids in the plant extracts, with flavonoids shielding lipids, proteins and DNA from oxidative damage [63]. However, further toxicological studies are required to verify the safety of these extracts before being considered for therapeutic uses.

A few studies have tested mountain pepper extracts in aquatic organisms. Rayan et al. (2017), Wright et al. (2017), and Aldosary et al. (2019) assessed their toxicological effects in *Artemia fransiscana* [58,62] and *Artemia nauplii* [23,62]. All extracts were non-toxic to both invertebrate models, with LC50 values substantially greater than 1000 µg/mL (or LC50 > 1000 µg/mL). In contrast, the LC50 value of mountain pepper extract calculated in zebrafish embryos was much lower (LC50-96h = 1.70 mg/L), which could be explained by differences in extracts and test conditions. For instance, these authors prepared the extracts using different solvents such as methanol, water, ethyl acetate, chloroform and hexane, and we used ethanol; the plant material and solvent proportion they used was 1 g:50 mL of solvent, and the extracts here were prepared using 1 g:10 mL of ethanol (70%); the medium used was seawater for the *Artemia spp.* assays and E3 medium for the zebrafish embryos; and the time of exposure was 72 h for *Artemia* spp. and 96 h for zebrafish. It remains critical to standardise these parameters for comparable studies and to correlate the preparation of extracts with toxicology for the future efficacy testing of the compounds.

The adverse effects and lethality observed after exposure to mountain pepper extracts were more frequent than to the finger lime and tamarind extracts. These effects could be attributed to the presence of eriodictyol 7-O-glucoside, estragole, and syringaresinol specifically identified in the mountain pepper extract (Table S1). The toxicity of these isolated compounds was previously reported in different models [64,65,66]. Singh et al. (2022) performed an in silico docking study of eriodictyol 7-O-glucoside observing AMES toxicity and carcinogenicity with poor intestinal absorption [64]. The authors suggested that this compound is not suitable in pharmaceutical formulations, not fulfilling the drug likeness and ADMET (absorption, distribution, metabolism, excretion, and toxicity) criteria. Estragole is a compound known to be toxic for *Drosophila melanogaster* [67], genotoxic and hepatocarcinogen, forming DNA adducts in rodent liver [65]. Kirsch et al. (2020) [66] predicted in silico that syringaresinol can act through different mechanisms such as cellular stress and chromosomal damage. However, in vitro assays in HepG2 and HT29 cells showed that no such toxicities (cytotoxicity, DNA damage and DNA strand breaks) were induced by physiological and higher concentrations of syringaresinol [66]. 

It is important to mention that the toxic effects observed in zebrafish larvae exposed to mountain pepper, finger lime and tamarind extracts could also be explained by the interactions of the compounds present in their extracts leading to the altered potentiation of individual compounds. The toxicity of plant extracts and their isolated compounds could limit their relevance for pharmaceutical applications. Thus, it is necessary to assess the risk of the compounds identified in these plants using different preclinical models.

## 4. Conclusions

Despite the incredible diversity of native Australian plants, the detailed screening of their bioactive metabolites is still scarce. In this study, a total of 96 metabolites were identified using LC-ESI-QTOF-MS/MS. The combination and abundance diversity of these metabolites in different extracts might have potentially distinct benefits for different pathologies. Continued systematic comparison will, in future, allow us to start correlating which compounds influence LC50 most strongly and drive unwanted toxic effects, which compounds are particularly bioactive and provide high antioxidant activity in vivo, and which combinations synergistically support health benefits. Future work based on such knowledge will support the separation, purification and more targeted combination of metabolites whilst reducing toxic effects. Our established rapid in vivo screening pipeline supported by in-depth biochemical characterisation will facilitate the assessment and optimisation of unique combinations by allowing us to assess biological activities, interactions, and utility towards clinical translation. This study presents a comparative framework towards identifying, assessing, and optimising the safe and effective use of plant resources as therapeutics.

## Figures and Tables

**Table 1:** Estimation of total phenolic content (TPC) and antioxidant activities of finger lime, mountain pepper and tamarind.

Variables	TPC (mg GAE/g)	DPPH (mg TE/g)	ABTS (mg TE/g)	RPA (mg TE/g)
Finger lime	0.71 ± 0.00 ^c^	0.44 ± 0.06 ^b^	0.92 ± 0.05 ^b^	0.69 ± 0.04 ^c^
Mountain pepper	5.91 ± 0.32 ^a^	4.48 ± 0.03 ^a^	6.68 ± 0.54 ^a^	13.23 ± 0.17 ^a^
Tamarind	3.72 ± 0.12 ^b^	4.07 ± 0.00 ^a^	6.86 ± 0.35 ^a^	12.52 ± 0.46 ^b^

The results per gram of sample are presented as mean ± standard deviation (*n* = 3). Superscript letters (^a–c^) indicate significantly different groups (Tukey’s honestly significant difference (HSD) multiple rank test at *p* < 0.05). DPPH—2,2′-diphenyl-1-picrylhydrazyl assay. RPA—reducing power assay; ABTS—2,2′-azino-bis-3-ethylbenzothiazoline-6-sulfonic acid assay. GAE—Gallic acid equivalent; TE—Trolox Equivalent.

## Data Availability

The data are within the article and the Supplementary Material.

## Supplementary Materials

The following supporting information can be downloaded at: https://www.mdpi.com/article/10.3390/antiox11071280/s1, Figure S1: Base peak chromatogram (BPC) of finger lime (A,D), mountain pepper (B,E) and tamarind (C,F) in negative (red colour) and positive (black colour) mode; Figure S2: LC-ESI-QTOF-MS/MS identification of cinnamic acid. Chromatogram (A) and a mass spectrum (B) obtained for mountain pepper in negative mode. The MS/MS product ion mass spectra were confirmed through the LC-MS library and database (C). Cinnamic acid produced mass spectra at *m/z 103* after the loss of CO_2_ from precursor ion (D); Figure S3: Chromatogram and mass spectrum of some selected compounds obtained by LC-MS/MS; Table S1: LC-ESI-MS/MS-QTOF metabolites profiling of finger lime, mountain pepper and tamarind; Table S2: HPLC-PDA quantification of targeted phenolic compounds (μg/g) in plant extracts.
