# Supplementary material for: Phytochemical and Safety Evaluations of Finger Lime, Mountain Pepper, and Tamarind in Zebrafish Embryos
Source: Antioxidants (Basel). 2022 Jun 28;11(7):1280. doi: 10.3390/antiox11071280 (PMC9311898; doi:10.3390/antiox11071280)
Supplement: Supplementary file 1 [file antioxidants-11-01280-s001.zip › antioxidants-1786849-supplementary.pdf]

antioxidants

MDPI

Supplementary Material

Phytochemical and safety evaluations of finger lime, mountain pepper, and tamarind in zebrafish embryos

Paolin Rocio Cáceres-Vélez [1,*], Akhtar Ali [2], Alexandre Fournier-Level[1], Frank R. Dunshea [2], Patricia Regina Jusuf [1,*]

[1] School of Biosciences, The University of Melbourne, Parkville VIC 3010, Australia; alexandre.fournier@unimelb.edu.au (AFL)
[2] School of Agriculture and Food, The University of Melbourne, Parkville VIC 3010, Australia; akali@student.unimelb.edu.au (AA); fdunshea@unimelb.edu.au (FRD)
* Correspondence: patricia.jusuf@unimelb.edu.au (PRJ); paolin.caceresvelez@unimelb.edu.au (PRCV)

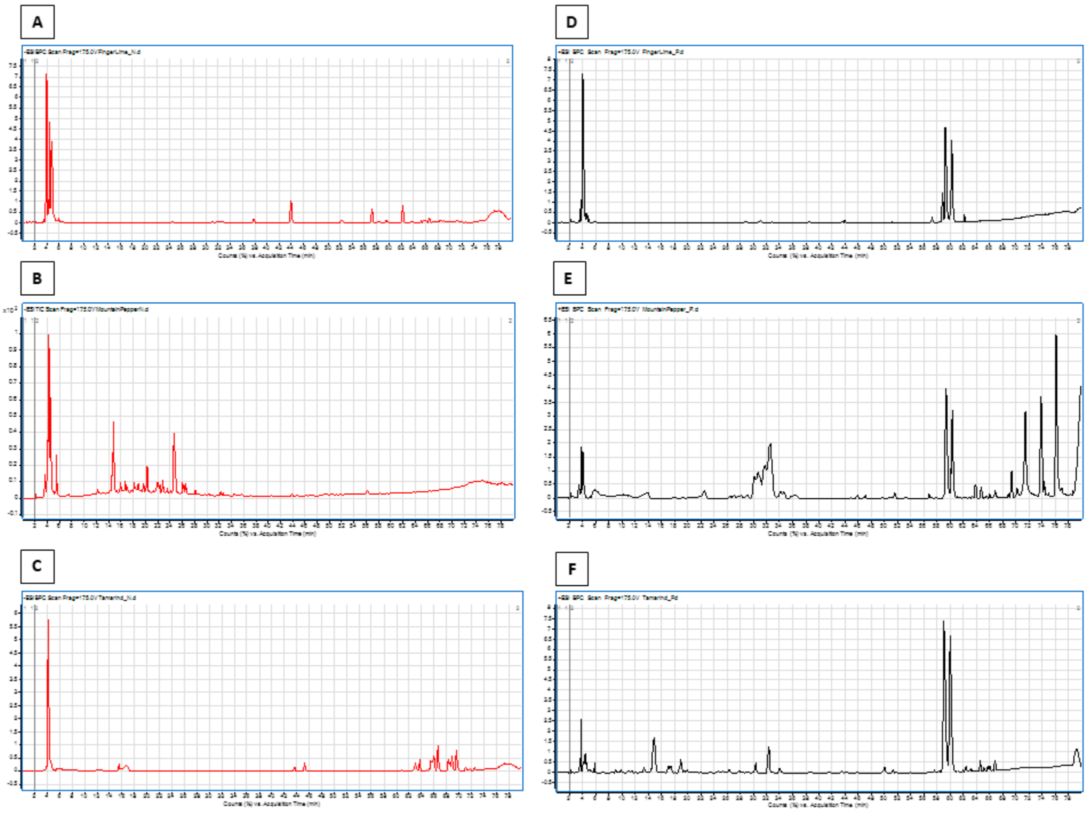

Figure S1. Base Peak Chromatogram (BPC) of Finger Lime (A, D), Mountain Pepper (B, E) and Tamarind (C, F) in Negative (Red Color) and Positive (Black color) mode.

Table S1. LC-ESI-MS/MS-QTOF phytochemical profiling of finger lime, mountain pepper and tamarind

No.	Proposed compounds	Molecular Formula	RT (min)	Mode of ionization	Theoretical (*m/z*)	Observed (*m/z*)	Mass Error (*ppm*)	Fragments	Samples
	**Phenolic Acids**								
	**Hydroxybenzoic acid**								
1	*p*-Hydroxybenzoic acid	$C_7H_6O_3$	4.131	$[M-H]^-$	137.0244	137.0220	-3.9	93, 65	T
2	* 2-Hydroxybenzoic acid	$C_7H_6O_3$	4.489	** $[M-H]^-$	137.0244	137.0244	0.0	93	MP, T, FL
3	* Gallic acid	$C_7H_6O_5$	5.401	[M-H]-	169.0142	169.0128	-8.3	125	MP, T
4	Gallic acid 4-*O*-glucoside	$C_{13}H_{16}O_{10}$	5.447	** $[M-H]^-$	331.0670	331.0670	0.0	169, 125	MP, T
5	Protocatechuic acid 4-*O*-glucoside	$C_{13}H_{16}O_9$	10.059	** $[M-H]^-$	315.0721	315.0731	3.2	153	MP, FL
6	Benzoic acid	$C_7H_6O_2$	10.698	** $[M-H]^-$	121.0295	121.0301	5.0	93, 77	MP, T
7	* Protocatechuic acid	$C_7H_6O_4$	10.986	$[M-H]^-$	153.0193	153.0182	-7.2	109	T
	**Hydroxycinnamic acids**								
8	Caffeic acid 3-*O*-glucuronide	$C_{15}H_{16}O_{10}$	3.687	$[M-H]^-$	355.0670	355.0662	-2.3	179	FL
9	Ferulic acid 4-*O*-glucuronide	$C_{16}H_{18}O_{10}$	4.035	$[M-H]^-$	369.0827	369.0811	-4.3	193	T
10	Dihydroferulic acid 4-*O*-glucuronide	$C_{16}H_{20}O_{10}$	5.447	$[M-H]^-$	371.0983	371.0968	-4.0	191, 164, 93	MP
11	*p*-Coumaroyl quinic acid	$C_{16}H_{18}O_8$	5.517	$[M-H]^-$	337.0929	337.0946	5.0	191, 179, 149, 119, 89	MP
12	Caffeic acid 4-*O*-glucoside	$C_{15}H_{18}O_9$	12.265	$[M-H]^-$	341.0878	341.0879	0.3	179, 161, 135	MP, FL
13	1-*O*-sinapoyl-*β*-*D*-glucose	$C_{17}H_{22}O_{10}$	13.526	$[M-H]^-$	385.1139	385.1097	-10.9	223, 205, 190	FL

14	*p*-Coumaric acid 4-*O*-glucoside	$C_{15}H_{18}O_8$	14.514	[M-H]$^-$	325.0929	325.0911	-4.7	163, 119	MP, FL
15	* *m*-Coumaric acid	$C_9H_8O_3$	14.621	** [M-H]$^-$	163.0400	163.0401	0.6	119	FL, MP, T
16	* 5-Caffeoylquinic acid	$C_{16}H_{18}O_9$	14.732	** [M-H]$^-$	353.0878	353.0885	2.0	191	MP, FL, T
17	Ferulic acid 4-*O*-glucoside	$C_{16}H_{20}O_9$	15.620	[M-H]$^-$	355.1034	355.1032	-0.6	193, 178, 149, 134	MP
18	* Caffeic acid	$C_9H_8O_4$	18.805	[M-H]$^-$	179.0496	179.0505	5.0	161, 135	MP, T, FL
19	3-Feruloylquinic acid	$C_{17}H_{20}O_9$	20.061	[M-H]$^-$	367.1034	367.1037	0.8	193, 134	MP
20	* Cinnamic acid	$C_9H_8O_2$	27.944	** [M-H]$^-$	147.0451	147.0458	4.8	103, 77	MP, T
21	* Ferulic acid	$C_{10}H_{10}O_4$	39.241	[M-H]$^-$	193.0506	193.0504	-1.0	178, 149, 134	FL, MP
22	1,5-Dicaffeoylquinic acid	$C_{25}H_{24}O_{12}$	59.477	[M-H]$^-$	515.1195	515.1177	-3.9	353, 335, 191, 179	MP, FL
	**Hydroxyphenylpropanoic acid**								
23	Dihydroferulic acid 4-sulfate	$C_{10}H_{12}O_7S$	4.694	** [M-H]$^-$	275.0231	275.0239	2.9	195, 151, 177	MP, FL
24	3-Hydroxy-3-(3-hydroxyphenyl)propionic acid	$C_9H_{10}O_4$	19.180	[M-H]$^-$	181.0506	181.0512	3.3	163, 135, 119	MP
	**Hydroxyphenylacetic acid**								
25	Homovanillic acid	$C_9H_{10}O_4$	36.799	[M-H]$^-$	182.0579	181.0473	3.34	121	T
	**Flavonoids**								
	**Anthocyanins**								
26	Petunidin 3-*O*-glucoside	$C_{22}H_{23}O_{12}$	13.427	[M]+	479.1190	479.1198	1.7	317, 85	T
27	Cyanidin aldopentose hexoside	$C_{26}H_{29}O_{15}$	14.786	[M]+	581.1506	581.1507	0.2	582, 288, 287	T
28	Cyanidin 3-*O*-glucoside	$C_{21}H_{21}O_{11}$	15.442	[M]+	449.1084	449.1079	-1.1	341, 322, 288, 287	T, MP

29	Cyanidin	$C_{15}H_{11}O_6$	15.962	** [M]-	287.0556	287.0560	1.4	270, 242, 207	MP, T
30	Cyanidin-aldodipentoside	$C_{25}H_{27}O_{14}$	18.156	[M]+	551.1401	551.1418	3.1	288, 287	T
31	Pelargonidin 3-*O*-arabinoside	$C_{20}H_{19}O_9$	19.739	[M]+	403.1029	403.1026	-0.7	273	T
32	Delphinidin 3-*O*-sambubioside	$C_{26}H_{29}O_{16}$	22.423	[M]+	597.1456	597.1453	-0.5	303	T
33	Pelargonidin	$C_{15}H_{11}O_5$	24.380	[M]-	271.0606	271.0603	-1.1	272, 253, 225, 209,	MP
	**Flavanols**								
34	* Catechin 3-glucoside	$C_{21}H_{24}O_{11}$	15.815	** [M-H]$^-$	451.1246	451.1235	-2.4	407, 351, 275, 225, 191	MP, FL
35	* (+)-Catechin	$C_{15}H_{14}O_6$	15.231	** [M-H]$^-$	289.0717	289.0725	2.8	245	MP, T, FL
36	Epicatechin 3'-*O*-glucuronide	$C_{21}H_{22}O_{12}$	18.024	[M-H]$^-$	465.1038	465.1031	-1.5	371, 324, 308, 231	MP
37	Procyanidin trimer C1	$C_{45}H_{38}O_{18}$	15.559	[M+H]$^+$	867.2131	867.2156	2.9	715, 579, 409, 289	T
38	Cinnamtannin A2	$C_{60}H_{50}O_{24}$	20.462	[M+H]$^+$	1155.2765	1155.2790	2.2	867, 577, 409, 289	T
39	* Procyanidin dimer B2	$C_{30}H_{26}O_{12}$	23.307	** [M-H]$^-$	577.1351	577.1331	-3.5	451, 407, 289, 245	T
	**Flavanones**								
40	Narirutin	$C_{27}H_{32}O_{14}$	3.740	[M-H]$^-$	579.1719	579.1712	-1.2	271, 151	MP, T, FL
41	Neoeriocitrin	$C_{27}H_{32}O_{15}$	14.283	[M-H]$^-$	595.1668	595.1668	0.0	431, 287	MP
42	Eriodictyol 7-*O*-glucoside	$C_{21}H_{22}O_{11}$	15.962	[M-H]$^-$	449.1089	449.1100	2.4	287, 175, 151, 135	MP
43	Naringenin	$C_{15}H_{12}O_5$	18.877	** [M-H]-	273.0758	273.0761	1.1	177, 151, 119, 107	MP, FL, T
44	Naringenin 7-*O*-glucoside	$C_{21}H_{22}O_{10}$	21.809	[M-H]$^-$	433.1140	433.1153	3.0	373, 343, 303	MP
45	Naringin 6'-malonate	$C_{30}H_{34}O_{17}$	24.707	[M-H]$^-$	665.1723	665.1703	-3.0	384, 248, 179	MP
46	Poncirin	$C_{28}H_{34}O_{14}$	31.264	[M-H]-	593.1876	593.1901	4.2	473, 327, 285, 270	FL

47	Brutieridin	$C_{34}H_{42}O_{18}$	43.605	$[M-H]^-$	737.2298	737.2226	-9.7	676, 636, 594, 286	FL
	**Flavones**								
48	5,6-Dihydroxy-7,8,3',4'-tetramethoxyflavone	$C_{19}H_{18}O_8$	4.368	$[M-H]^-$	373.0929	373.0920	-2.4	342, 274, 217, 93	MP
49	Apigenin 6,8-di-C-glucoside	$C_{27}H_{30}O_{15}$	15.521	** [M-H]-	593.1512	593.1511	-0.2	503, 473	MP, FL, T
50	Apigenin 6-C-glucoside	$C_{21}H_{20}O_{10}$	20.592	$[M-H]^-$	431.0983	431.0974	-2.1	341, 311, 283, 163, 117	MP
51	* Luteolin	$C_{15}H_{10}O_6$	21.981	** $[M-H]^-$	285.0404	285.0400	-1.4	153	MP, T
52	Rhoifolin	$C_{27}H_{30}O_{14}$	22.706	** $[M-H]^-$	577.1563	577.1572	1.6	475, 431, 269	MP, T
	**Flavonols**								
53	Quercetin 3'-*O*-glucuronide	$C_{21}H_{18}O_{13}$	5.867	$[M-H]^-$	477.0674	477.0697	4.8	301	FL
54	Quercetin 3-*O*-xylosyl-rutinoside	$C_{32}H_{38}O_{20}$	11.420	$[M+H]^+$	743.2029	743.2014	-2.0	479, 317	T
55	Kaempferol 3,7,4'-*O*-triglucoside	$C_{33}H_{40}O_{21}$	13.129	$[M-H]^-$	771.1989	771.1995	0.8	609, 446, 285	MP, FL
56	* Rutin	$C_{27}H_{30}O_{16}$	20.203	$[M-H]^-$	609.1461	609.1444	-2.8	301	MP, FL
57	Myricetin-3-*O*-rhamnoside	$C_{21}H_{20}O_{12}$	21.598	** $[M-H]^-$	463.0882	463.0887	1.1	317	MP, FL, T
58	Isorhamnetin 3-*O*-rutinoside	$C_{28}H_{32}O_{16}$	22.146	** $[M-H]^-$	623.1617	623.1618	0.2	314	MP, FL
59	Quercetin 3-*O*-arabinoside	$C_{20}H_{18}O_{11}$	22.813	$[M-H]^-$	433.0776	433.0776	0.0	301	MP
60	Kaempferol 3-*O*-(2''-rhamnosyl-galactoside) 7-*O*-rhamnoside	$C_{33}H_{40}O_{19}$	24.707	$[M-H]^-$	739.2091	739.2084	-0.9	593, 447, 285	MP
61	Isorhamnetin	$C_{16}H_{12}O_7$	33.882	$[M-H]^-$	315.0510	315.0498	-3.8	300	FL
	**Isoflavonoids**								

62	6''-*O*-Acetyldaidzin	$C_{23}H_{22}O_{10}$	3.832	$[M-H]^-$	457.1140	457.1144	0.9	221	FL
63	6''-*O*-Acetylglycitin	$C_{24}H_{24}O_{11}$	27.308	$[M+H]^+$	489.1392	489.1376	-3.3	285, 270	FL, T
64	2',7-Dihydroxy-4',5'-dimethoxyisoflavone	$C_{17}H_{14}O_6$	14.448	$[M-H]^-$	313.0717	313.0723	1.9	283	MP
65	3',4',5,7-Tetrahydroxyisoflavanone	$C_{15}H_{12}O_6$	15.962	** $[M-H]^-$	287.0561	287.0562	0.3	259, 217, 179, 151, 109	MP, T
66	3′-Hydroxydaidzen	$C_{15}H_{10}O_5$	24.659	** $[M-H]^-$	269.0455	269.0451	2.2	251, 225, 213, 181	MP, T
67	6''-*O*-Malonylgenistin	$C_{24}H_{22}O_{13}$	57.195	$[M-H]^-$	517.0982	517.0971	-2.1	271	FL
68	6''-*O*-Malonylglycitin	$C_{25}H_{24}O_{13}$	58.354	$[M-H]^-$	531.1139	531.1130	-1.6	285, 270, 253	FL, MP
	**Dihydroflavonols**								
69	Dihydroquercetin	$C_{15}H_{12}O_7$	21.624	$[M+H]^+$	305.0656	305.0654	-0.7	259, 231, 153	T
	**Other Polyphenols**								
	**Phenolic terpenes**								
70	Rosmadial	$C_{20}H_{24}O_5$	26.343	$[M-H]^-$	343.1551	343.1566	4.4	315	MP
	**Naphtoquinones**								
71	Juglone	$C_{10}H_6O_3$	4.420	$[M-H]^-$	173.02389	173.0231	-4.5	155	T
	**cylitols**								
72	Quinic Acid		3.869	$[M-H]^-$	191.0555	191.0551	-2.0	127, 85	FL, MP
	**Tyrosols**								
73	Tyrosol 4-sulfate	$C_8H_{10}O_5S$	76.059	** $[M-H]^-$	217.0175	217.0165	-4.6	137, 93	MP
	**Furanocoumarins**								
74	Isopimpinellin	$C_{13}H_{10}O_5$	58.742	** $[M+H]^+$	247.0606	247.0610	1.6	232, 217, 189, 161	FL, T

	**Hydroxycoumarins**								
75	Esculin	$C_{15}H_{16}O_9$	12.730	$[M-H]^-$	339.0721	339.0719	-0.6	177	MP
76	Mellein	$C_{10}H_{10}O_3$	12.950	$[M+H]^+$	179.0703	179.0700	-1.7	161, 155, 151, 133	T
77	Coumarin	$C_9H_6O_2$	15.231	** $[M+H]^+$	147.0441	147.0449	5.4	103, 91	MP, T
78	Umbelliferone	$C_9H_6O_3$	16.636	** $[M-H]^-$	161.0244	161.0249	3.1	133, 117, 105	MP, T
79	Scopoletin	$C_{10}H_8O_4$	62.103	$[M+H]^+$	193.0496	193.0504	4.1	176	T
	**Hydroxyphenylpropenes**								
80	Estragole	$C_{10}H_{12}O$	15.359	$[M+H]^+$	149.0961	149.0964	2.0	137, 131, 123	MP
	**Phenylethanoic acid**								
81	Phenylacetic acid	$C_8H_8O_2$	14.732	$[M-H]^-$	135.0451	135.0451	0.0	134, 107, 91	MP
	**Hydroxybenzoketones**								
82	Norathyriol	$C_{13}H_8O_6$	3.516	$[M-H]^-$	259.0248	259.0242	-2.3	230, 215, 187, 169	FL
83	2,3-Dihydroxy-1-guaiacylpropanone	$C_{10}H_{12}O_5$	26.480	$[M+H]^+$	213.0758	213.0764	2.8	167, 123, 105, 93	T
	**Hydroxybenzaldehydes**								
84	Vanillin	$C_8H_8O_3$	18.877	$[M+H]^+$	153.0546	153.0545	-0.7	125	T
	**Other polyphenols**								
85	* Carvacrol	$C_{10}H_{14}O$	14.960	[M+H]+	151.1118	151.1116	-1.3	133, 109, 107	MP
86	Catechol	$C_6H_6O_2$	4.420	** $[M-H]^-$	111.0441	109.0273	2.7	91, 65	MP, T
87	Phlorin	$C_{12}H_{16}O_8$	5.029	** $[M-H]^-$	287.0772	287.0781	3.1	271, 253, 163, 127	MP, FL

88	* Pyrogallol	$C_6H_6O_3$	10.867	$[M+H]^+$	127.0390	127.0390	0.0	109, 81	T
	**Stilbenes**								
89	4-Hydroxy-3,5,4'-trimethoxystilbene	$C_{17}H_{18}O_4$	3.757	$[M+H]^+$	287.1278	287.1282	1.4	271, 241, 225	FL, T
90	Dihydroresveratrol	$C_{14}H_{14}O_3$	14.345	$[M+H]^+$	231.1016	231.1027	4.8	137, 121, 107	MP
91	3,4,5,4'-Tetramethoxystilbene	$C_{26}H_{44}N_4O_2S$	18.735	$[M-H]^-$	475.3112	475.3102	-2.1	433, 279, 163, 89	MP
	**Lignans**								
92	Secoisolariciresinol	$C_{20}H_{26}O_6$	18.540	$[M-H]^-$	361.1656	361.1653	-0.8	165, 121	MP
93	Syringaresinol	$C_{22}H_{26}O_8$	21.981	** $[M+H]^+$	417.1555	417.1542	-3.1	401, 388, 369	MP
	**Non-phenolic metabolites**								
94	Succinic acid	$C_4H_6O_4$	5.164	$[M-H]^-$	117.0193	117.0171	2.28	99, 73	T
95	4-(Decan-4-yl)benzenesulfonic acid	$C_{16}H_{26}O_3S$	6.153	$[M-H]^-$	297.1529	297.1503	-8.7	183, 119, 79	T
	**Sesquiterpenoids**								
96	Curcumenol	$C_{15}H_{22}O_2$	71.304	$[M+H]^+$	235.1692	235.1737	4.5	217, 189, 133	MP

*= confirmed with pure standards, **= identified in both positive and negative mod

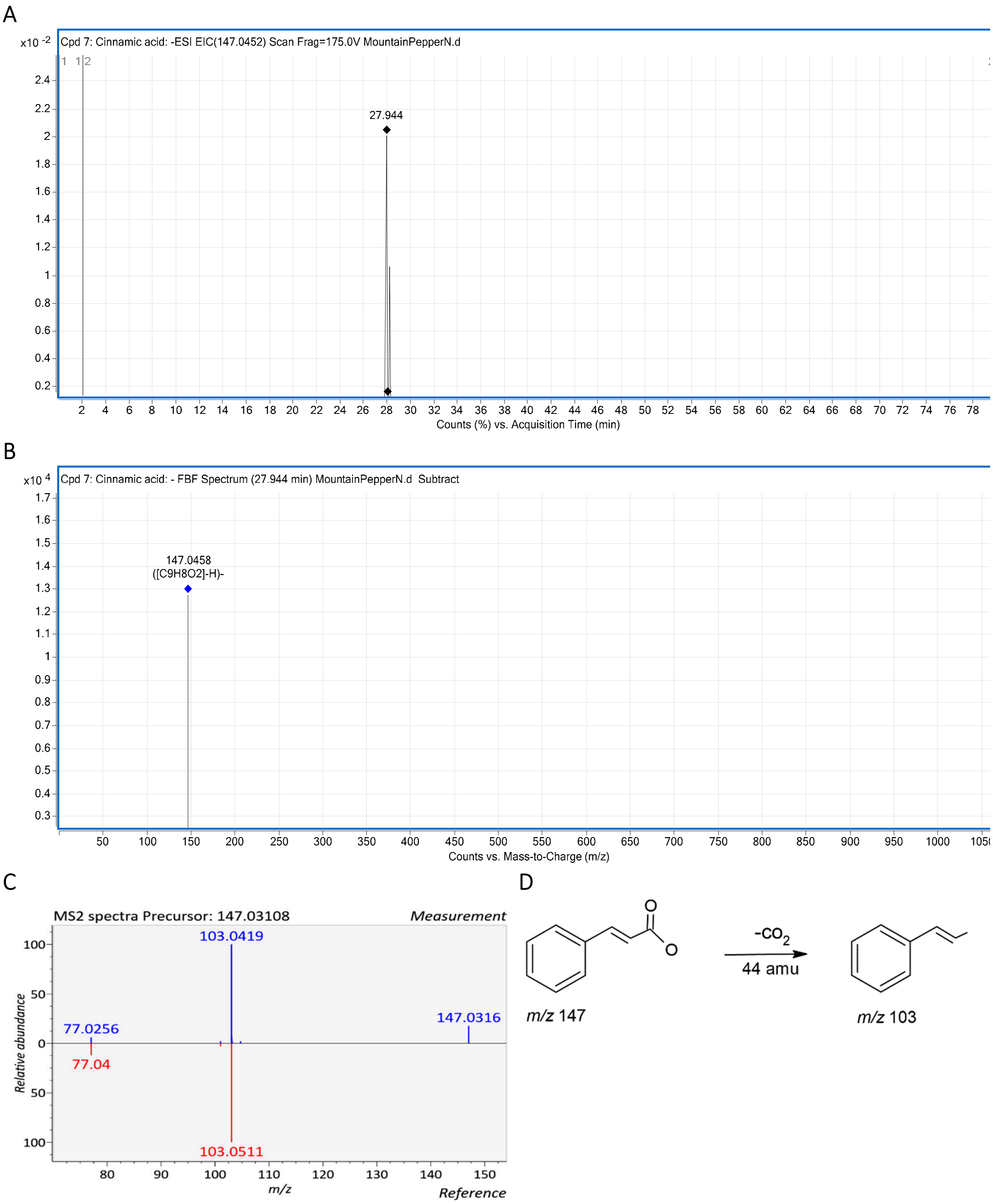
A
Cpd 7: Cinnamic acid: -ESI EIC(147.0452) Scan Frag=175.0V MountainPepperN.d
x10 -2
27.944
Counts (%) vs. Acquisition Time (min)
B
Cpd 7: Cinnamic acid: - FBF Spectrum (27.944 min) MountainPepperN.d Subtract
x10 4
147.0458
([C9H8O2]-H)-
Counts vs. Mass-to-Charge (m/z)
C
MS2 spectra Precursor: 147.03108
Measurement
103.0419
77.0256
147.0316
77.04
103.0511
Relative abundance
m/z
Reference
D
-CO2
44 amu
m/z 147
m/z 103

Figure S2. LC-ESI-QTOF-MS/MS identification of cinnamic acid. Chromatogram (A) and a mass spectrum (B) obtained for Mountain pepper in negative mode. The MS/MS product ion mass spectra were confirmed through the LC-MS library and database (C). Cinnamic acid produced mass spectra at *m/z 103* after the loss of CO_2 from precursor ion (D).

p-hydroxybenzoic acid

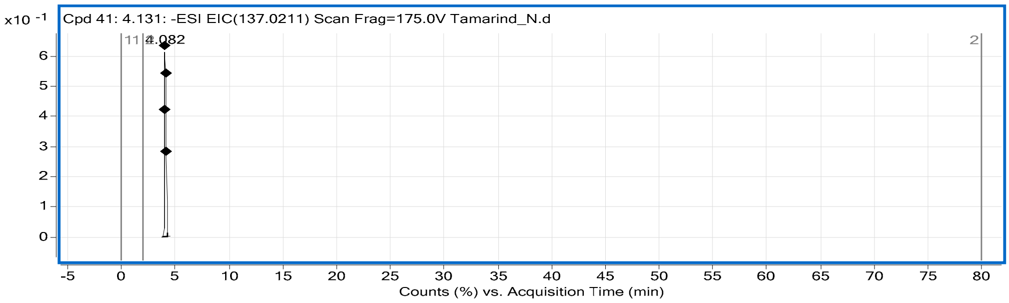

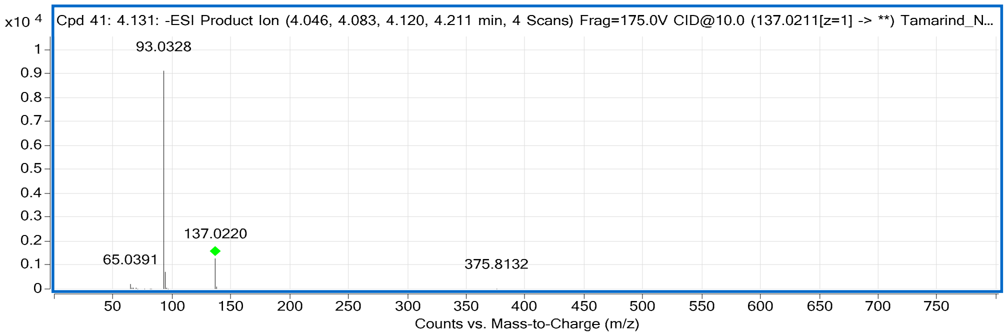

Protocatechuic acid

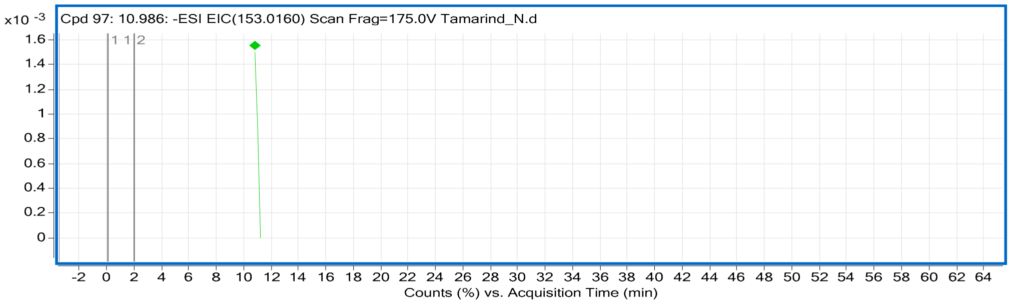

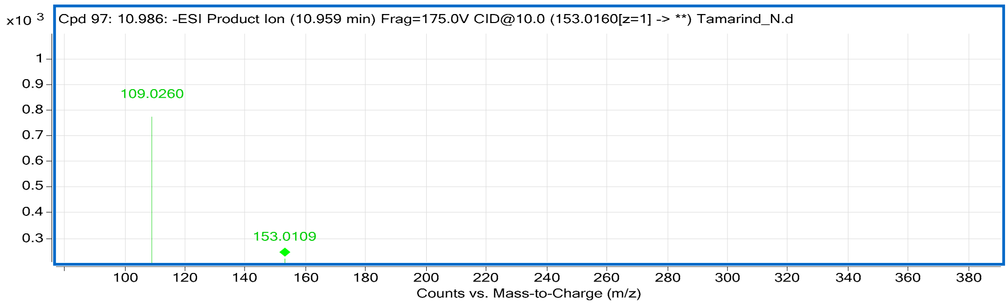

Gallic acid 4-*O*-glucoside

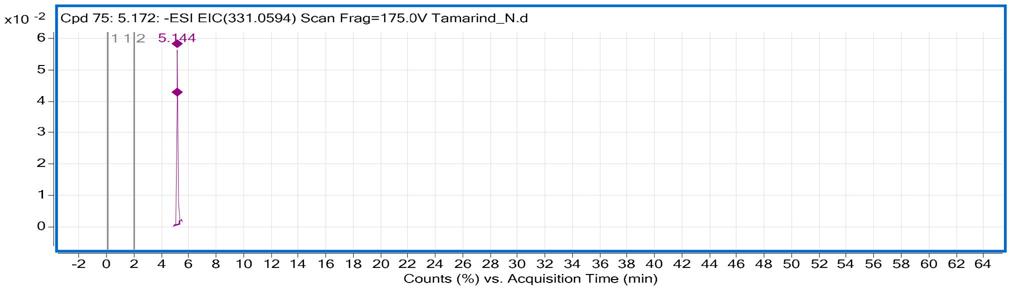

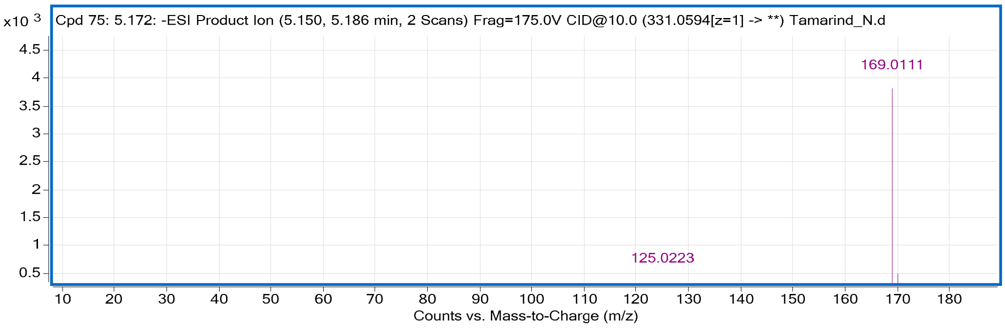

Cinnamic acid

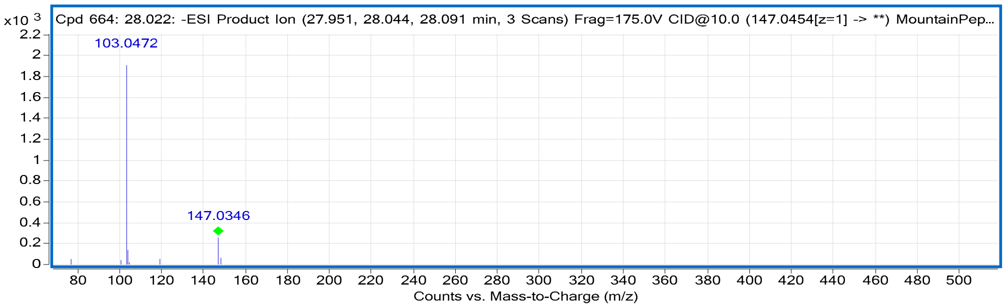

2-hydroxybenzoic acid

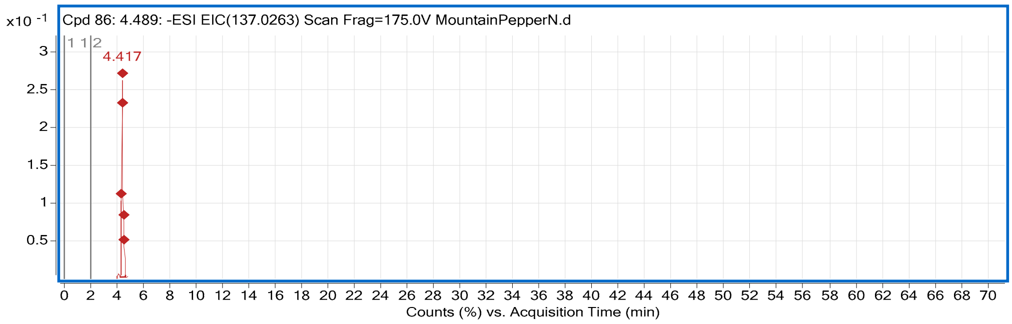

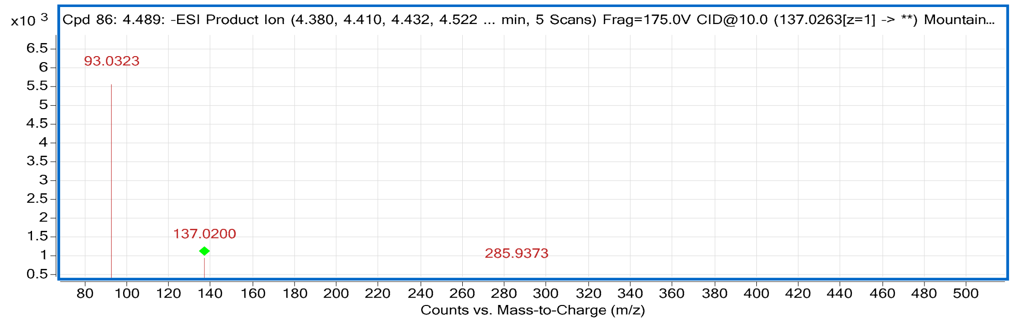

Coumaric acid

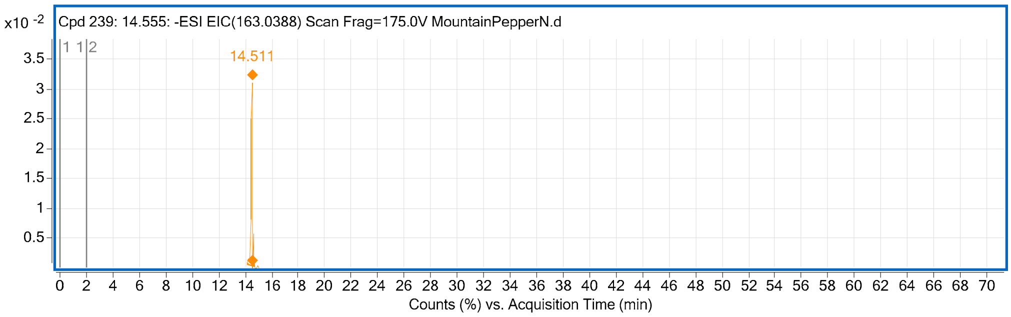

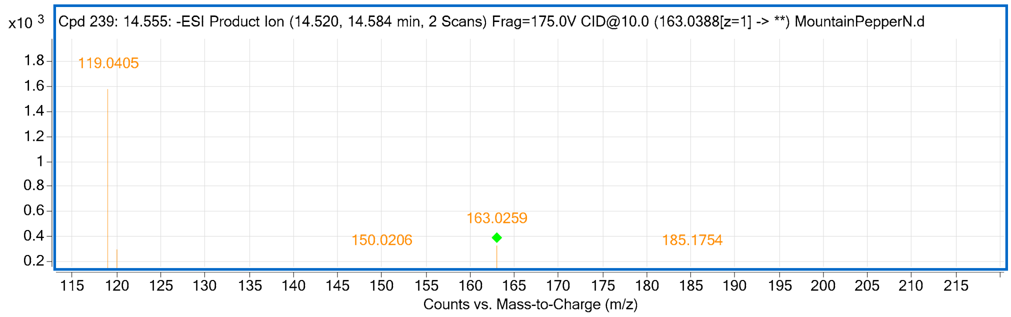

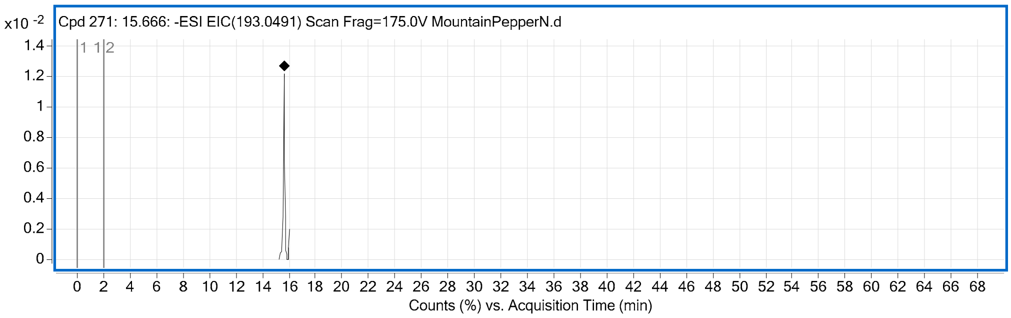

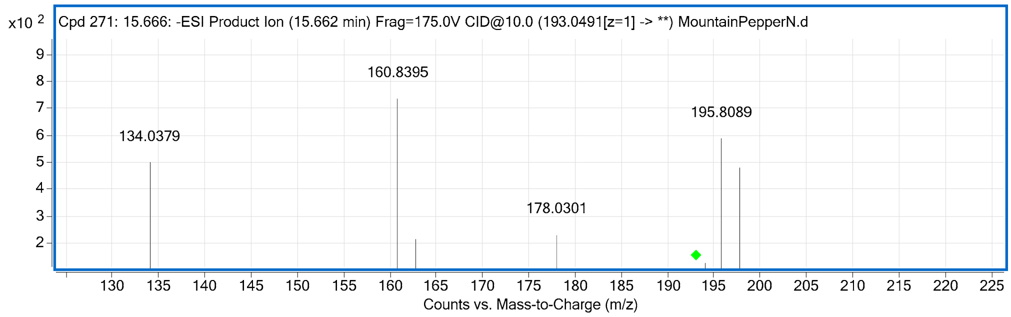

3-hydroxy-3-(3-hydrxyphenyl)propionic acid

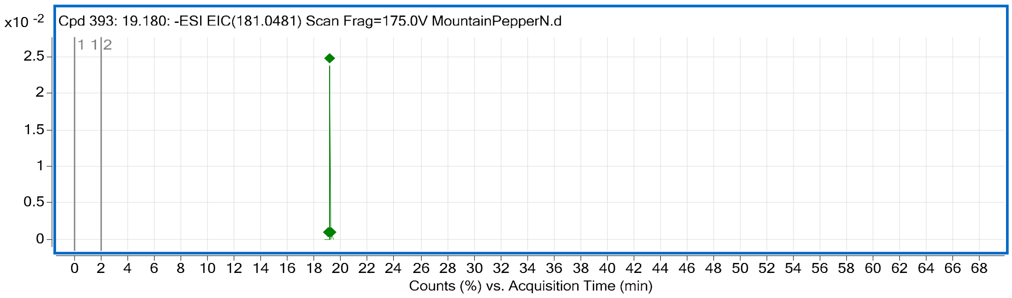

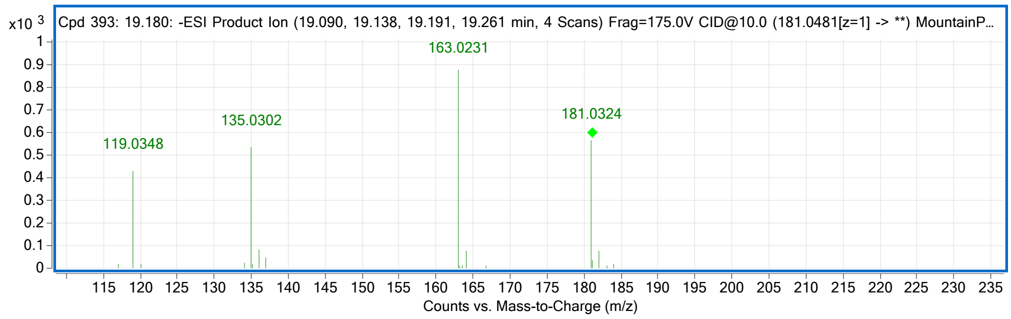

p-coumaric acid 4-*O*-glucoside

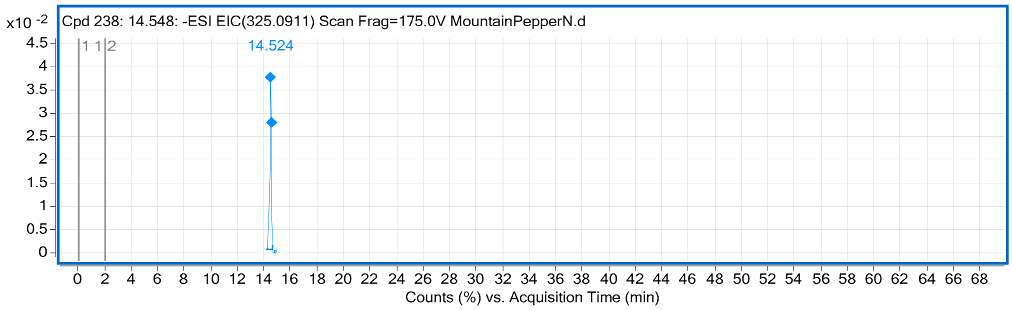
Cpd 238: 14.548: -ESI EIC(325.0911) Scan Frag=175.0V MountainPepperN.d
x10 -2
14.524
Counts (%) vs. Acquisition Time (min)

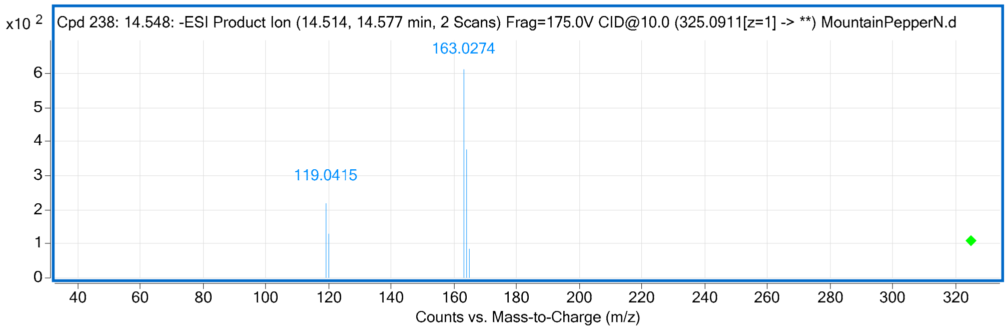
Cpd 238: 14.548: -ESI Product Ion (14.514, 14.577 min, 2 Scans) Frag=175.0V CID@10.0 (325.0911[z=1] -> **) MountainPepperN.d
x10 2
163.0274
119.0415
Counts vs. Mass-to-Charge (m/z)

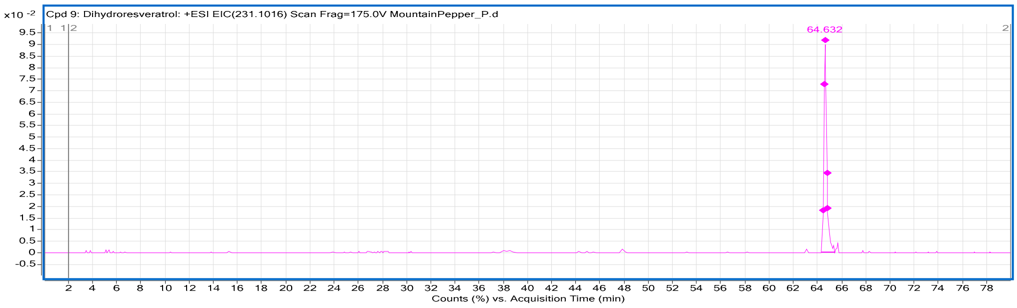
Cpd 9: Dihydroresveratrol: +ESI EIC(231.1016) Scan Frag=175.0V MountainPepper_P.d
x10 -2
64.632
Counts (%) vs. Acquisition Time (min)

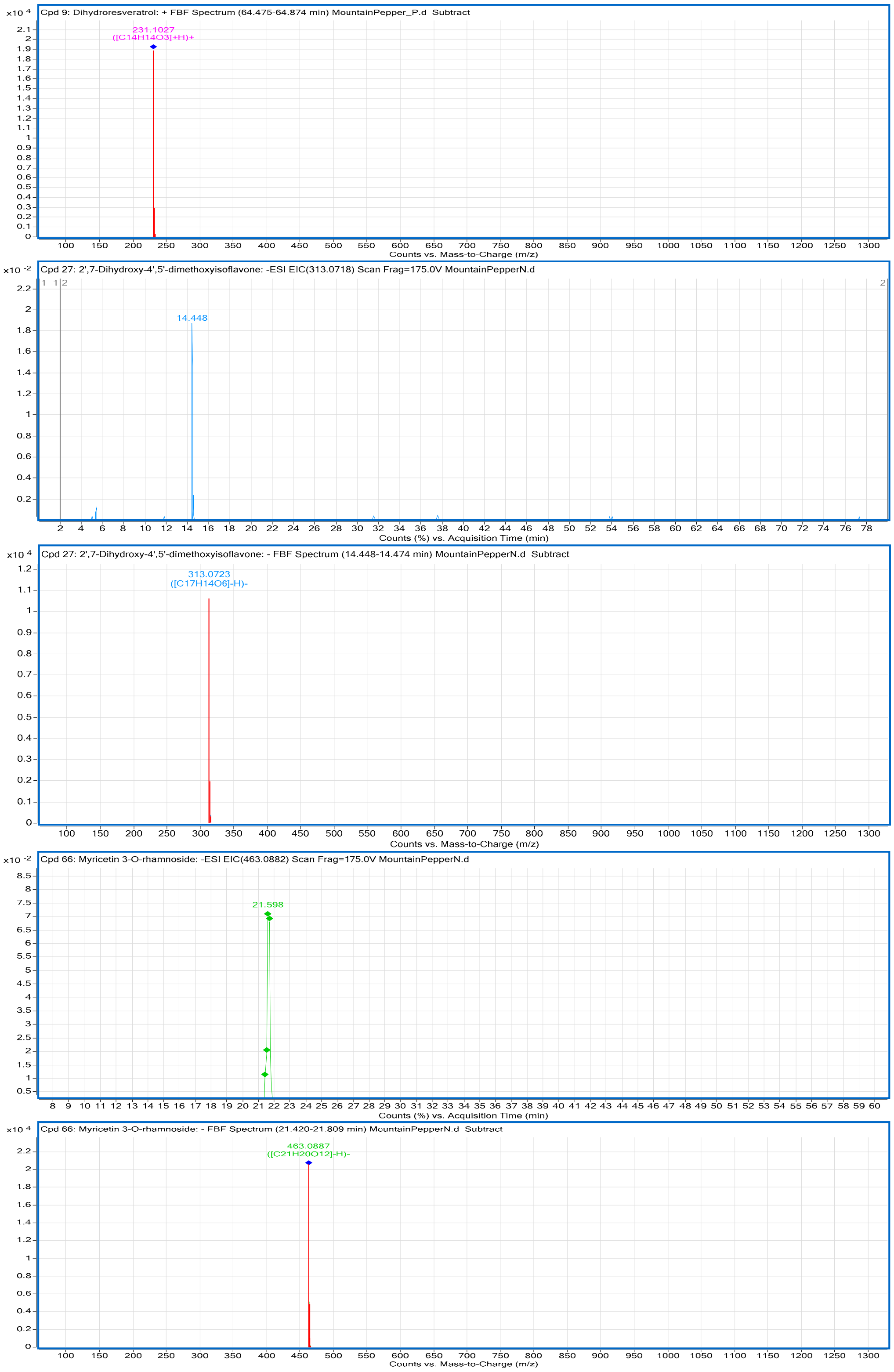
Cpd 9: Dihydroresveratrol: + FBF Spectrum (64.475-64.874 min) MountainPepper_P.d Subtract
231.1027
([C14H14O3]+H)+
Counts vs. Mass-to-Charge (m/z)
Cpd 27: 2',7-Dihydroxy-4',5'-dimethoxyisoflavone: -ESI EIC(313.0718) Scan Frag=175.0V MountainPepperN.d
14.448
Counts (%) vs. Acquisition Time (min)
Cpd 27: 2',7-Dihydroxy-4',5'-dimethoxyisoflavone: - FBF Spectrum (14.448-14.474 min) MountainPepperN.d Subtract
313.0723
([C17H14O6]-H)-
Counts vs. Mass-to-Charge (m/z)
Cpd 66: Myricetin 3-O-rhamnoside: -ESI EIC(463.0882) Scan Frag=175.0V MountainPepperN.d
21.598
Counts (%) vs. Acquisition Time (min)
Cpd 66: Myricetin 3-O-rhamnoside: - FBF Spectrum (21.420-21.809 min) MountainPepperN.d Subtract
463.0887
([C21H20O12]-H)-
Counts vs. Mass-to-Charge (m/z)

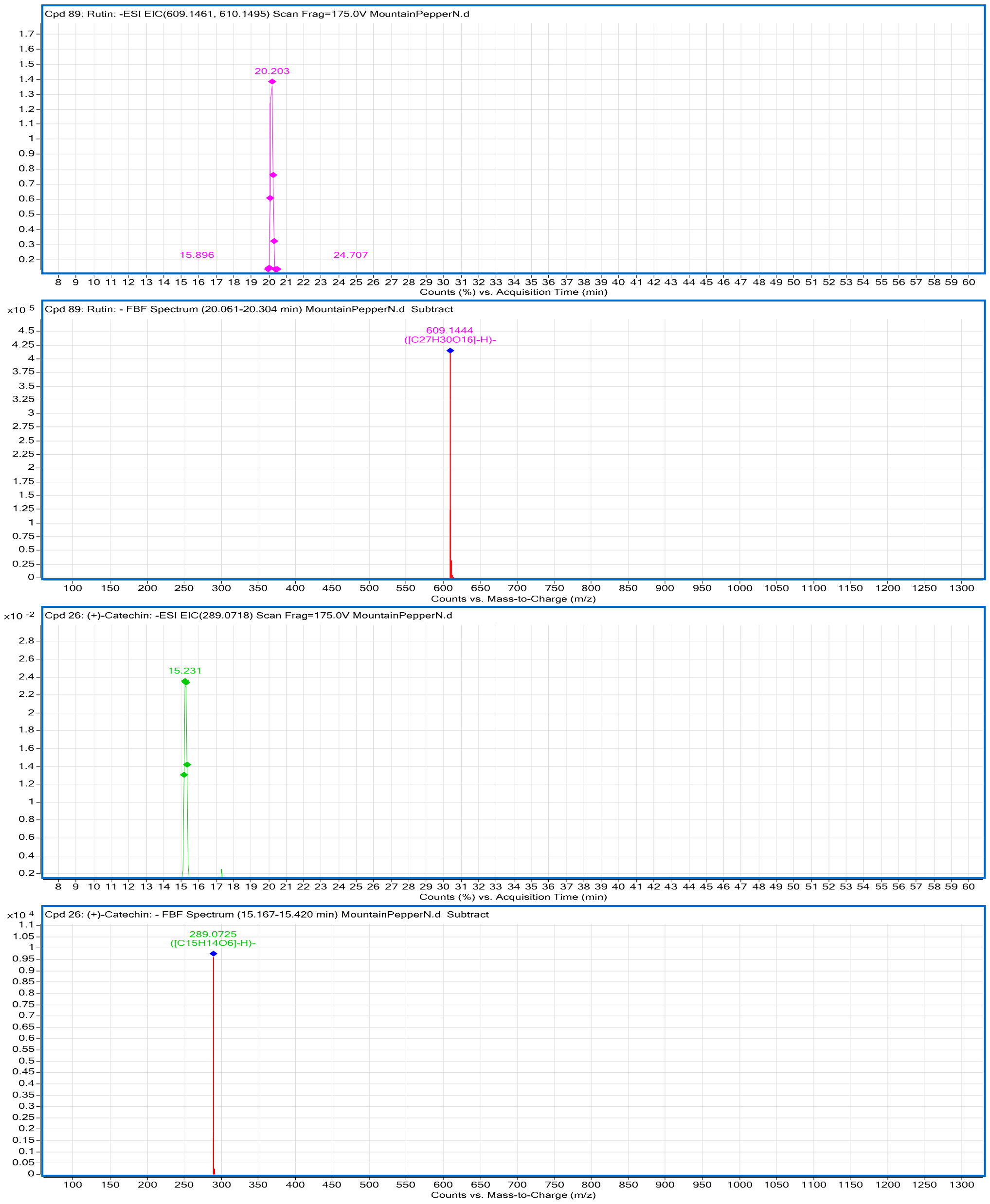
Cpd 89: Rutin: -ESI EIC(609.1461, 610.1495) Scan Frag=175.0V MountainPepperN.d
20.203
15.896
24.707
Counts (%) vs. Acquisition Time (min)
Cpd 89: Rutin: - FBF Spectrum (20.061-20.304 min) MountainPepperN.d Subtract
609.1444
([C27H30O16]-H)-
Counts vs. Mass-to-Charge (m/z)
Cpd 26: (+)-Catechin: -ESI EIC(289.0718) Scan Frag=175.0V MountainPepperN.d
15.231
Counts (%) vs. Acquisition Time (min)
Cpd 26: (+)-Catechin: - FBF Spectrum (15.167-15.420 min) MountainPepperN.d Subtract
289.0725
([C15H14O6]-H)-
Counts vs. Mass-to-Charge (m/z)

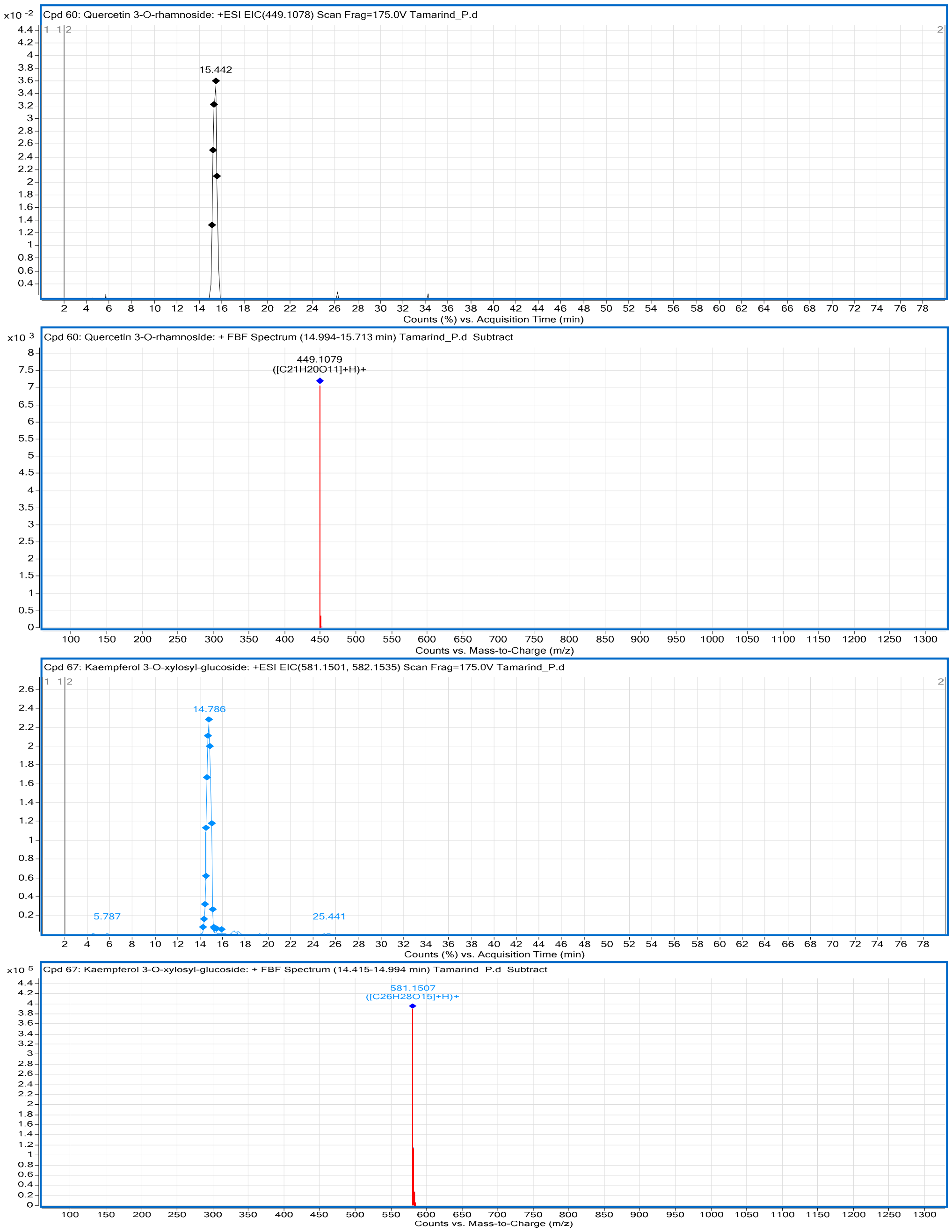
Cpd 60: Quercetin 3-O-rhamnoside: +ESI EIC(449.1078) Scan Frag=175.0V Tamarind_P.d
15.442
Counts (%) vs. Acquisition Time (min)
Cpd 60: Quercetin 3-O-rhamnoside: + FBF Spectrum (14.994-15.713 min) Tamarind_P.d Subtract
449.1079
([C21H20O11]+H)+
Counts vs. Mass-to-Charge (m/z)
Cpd 67: Kaempferol 3-O-xylosyl-glucoside: +ESI EIC(581.1501, 582.1535) Scan Frag=175.0V Tamarind_P.d
14.786
5.787
25.441
Counts (%) vs. Acquisition Time (min)
Cpd 67: Kaempferol 3-O-xylosyl-glucoside: + FBF Spectrum (14.415-14.994 min) Tamarind_P.d Subtract
581.1507
([C26H28O15]+H)+
Counts vs. Mass-to-Charge (m/z)

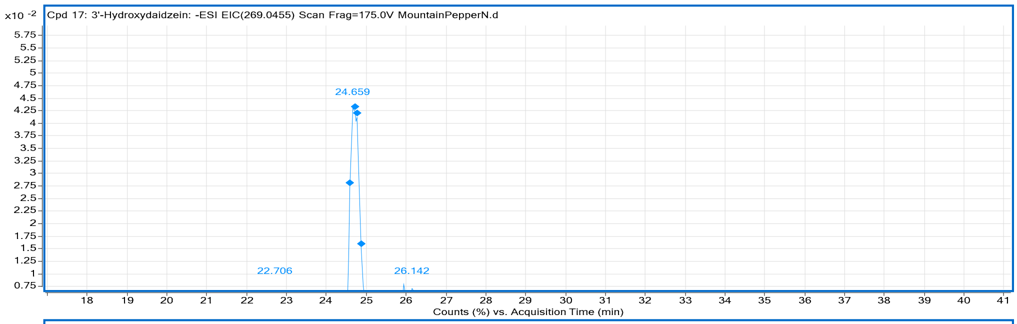
Cpd 17: 3'-Hydroxydaidzein: -ESI EIC(269.0455) Scan Frag=175.0V MountainPepperN.d
x10 -2
24.659
22.706
26.142
Counts (%) vs. Acquisition Time (min)

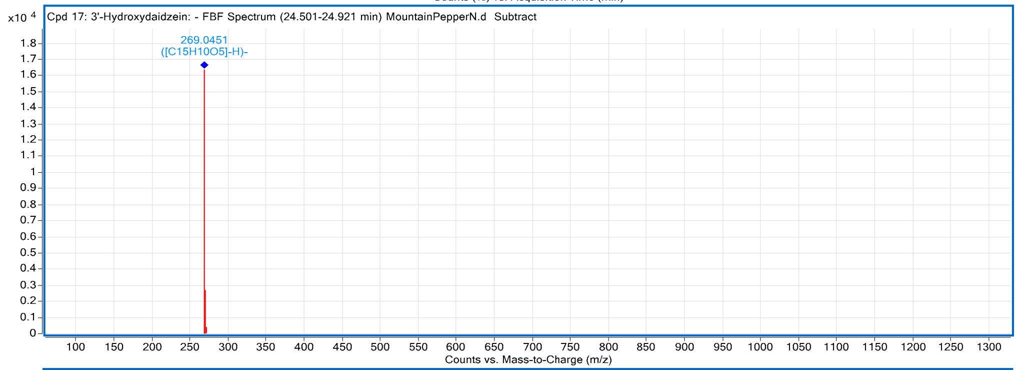
Cpd 17: 3'-Hydroxydaidzein: - FBF Spectrum (24.501-24.921 min) MountainPepperN.d Subtract
x10 4
269.0451
([C15H10O5]-H)-
Counts vs. Mass-to-Charge (m/z)

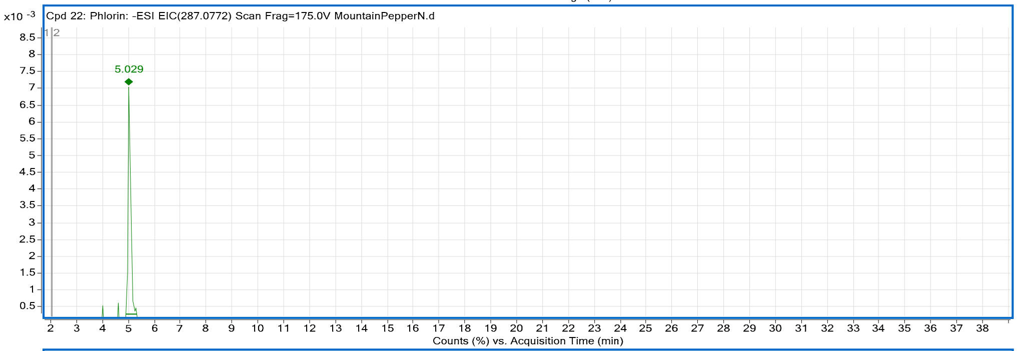
Cpd 22: Phlorin: -ESI EIC(287.0772) Scan Frag=175.0V MountainPepperN.d
x10 -3
5.029
Counts (%) vs. Acquisition Time (min)

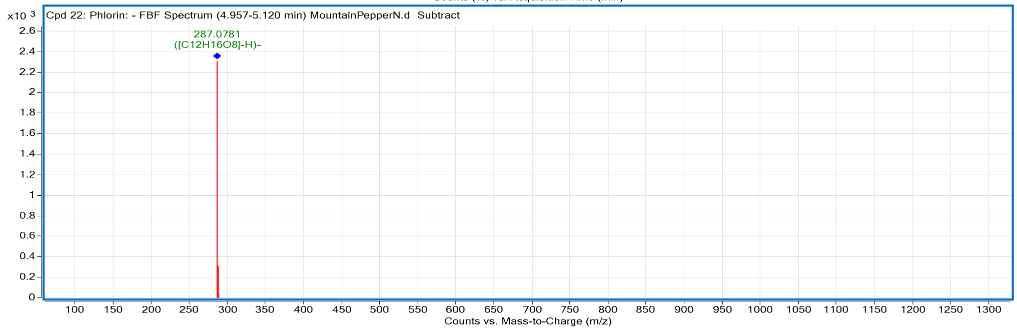
Cpd 22: Phlorin: - FBF Spectrum (4.957-5.120 min) MountainPepperN.d Subtract
x10 3
287.0781
([C12H16O8]-H)-
Counts vs. Mass-to-Charge (m/z)

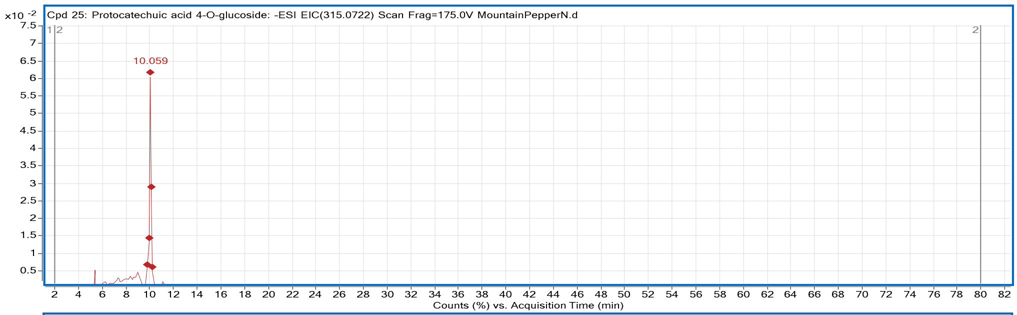
Cpd 25: Protocatechuic acid 4-O-glucoside: -ESI EIC(315.0722) Scan Frag=175.0V MountainPepperN.d
x10 -2
10.059
Counts (%) vs. Acquisition Time (min)

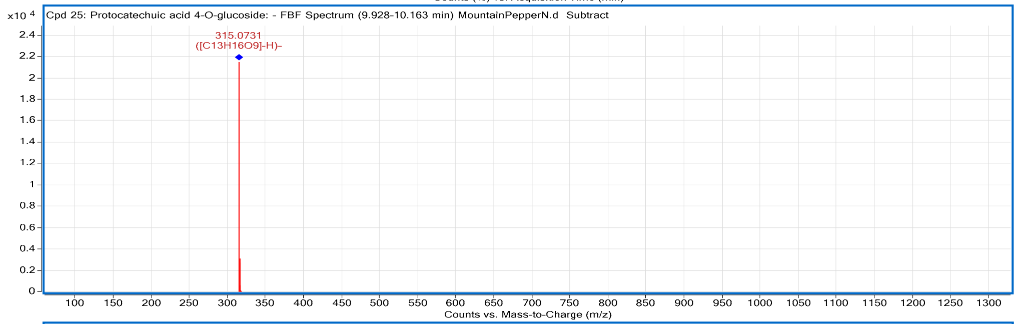
Cpd 25: Protocatechuic acid 4-O-glucoside: - FBF Spectrum (9.928-10.163 min) MountainPepperN.d Subtract
x10 4
315.0731
([C13H16O9]-H)-
Counts vs. Mass-to-Charge (m/z)

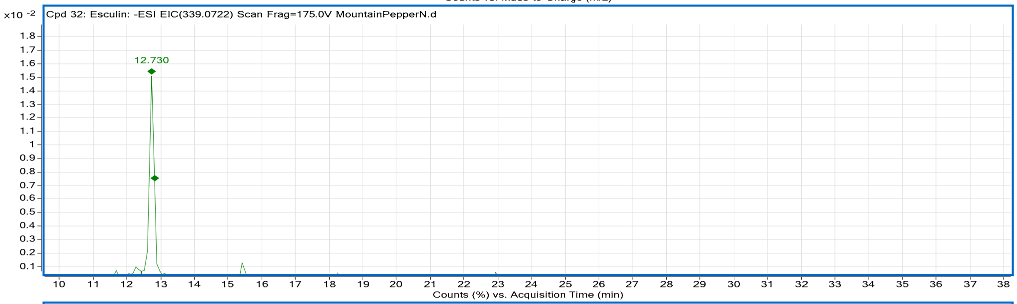
Cpd 32: Esculin: -ESI EIC(339.0722) Scan Frag=175.0V MountainPepperN.d
x10 -2
12.730
Counts (%) vs. Acquisition Time (min)

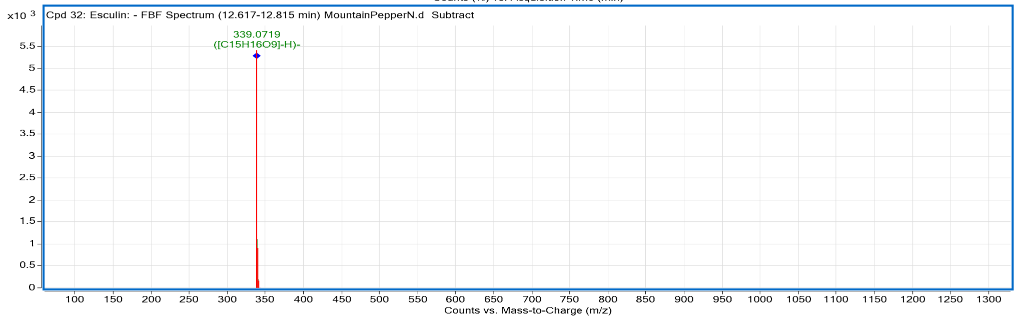
Cpd 32: Esculin: - FBF Spectrum (12.617-12.815 min) MountainPepperN.d Subtract
x10 3
339.0719
([C15H16O9]-H)-
Counts vs. Mass-to-Charge (m/z)

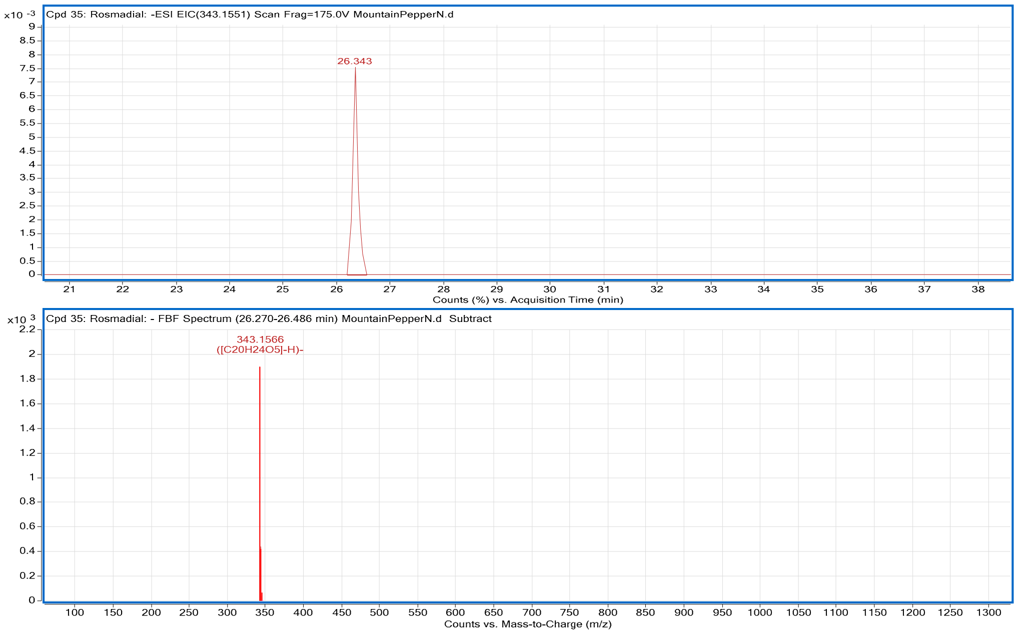

Figure S3. Chromatogram and Mass spectrum of some selected compounds obtained by LC-MS/MS.

Table S2. HPLC- PDA quantification of targeted phenolic compounds (µg/g) in plant extracts

No.	Compound Name	Formula	Finger Lime	Mountain pepper	Tamarind	Class
1	Gallic acid	$C_7H_6O_5$	NQ	21.80 ± 2.24 ^{de}	106.87 ± 14.93 ^{d}	PA
2	Protocatechuic acid	$C_7H_6O_4$	NQ	158.53 ± 47.61 ^{bc}	51.29 ± 6.87 ^{e}	PA
3	5-caffeoylquinic acid	$C_{16}H_{18}O_9$	132.91 ± 15.95 ^{a}	654.41 ± 43.44 ^{a}	327.81 ± 54.81 ^{a}	PA
4	Caffeic acid	$C_9H_8O_4$	77.38 ± 15.25 ^{c}	13.93 ± 3.81 ^{e}	299.39 ± 33.15 ^{b}	PA
5	Coumaric acid	$C_9H_8O_3$	4.08 ± 0.73 ^{e}	1.93 ± 0.04 ^{f}	51.56 ± 5.85 ^{e}	PA
6	Ferulic acid	$C_{10}H_{10}O_4$	75.95 ± 14.00 ^{c}	13.18 ± 1.57 ^{e}	NQ	PA
7	1,5-Dicaffeoylquinic acid	$C_{25}H_{24}O_{12}$	NQ	134.73 ± 9.02 ^{c}	NQ	PA
8	3-Feruloylquinic acid	$C_{17}H_{20}O_9$	NQ	123.38 ± 5.63 ^{c}	NQ	PA
9	Catechin 3-glucoside	$C_{21}H_{24}O_{11}$	NQ	15.88 ± 1.44 ^{e}	NQ	FN
10	Catechin	$C_{15}H_{14}O_6$	18.34 ± 1.77 ^{d}	22.86 ± 0.89 ^{de}	141.75 ± 9.29 ^{c}	FN
11	Luteolin	$C_{15}H_{10}O_6$	NQ	15.62 ± 1.34 ^{e}	47.20 ± 8.69 ^{ef}	FN
12	Carvacrol	$C_{10}H_{14}O$	NQ	15.10 ± 0.05 ^{e}	NQ	T
13	Pyrogallol	$C_6H_6O_3$	NQ	NQ	35.14 ± 8.73 ^{f}	OP
14	PCB2	$C_{30}H_{26}O_{12}$	NQ	NQ	41.02 + 5.93 ^{ef}	FN
15	2-hydroxybenzoic acid	$C_7H_6O_3$	106.68 ± 23.71 ^{b}	NQ	107.35 + 21.59 ^{d}	PA

PA= Phenolic acid, FN= Flavonoids, OP=Other polyphenols, T= Terpenoid, NQ=Not quantified.

Superscripted letters ($^{a-f}$) represent significant differences from each other.
